# Cerebrospinal fluid catecholamines in delirium and dementia

**DOI:** 10.1093/braincomms/fcab121

**Published:** 2021-05-29

**Authors:** Kristi Henjum, Kristin Godang, Else Quist-Paulsen, Ane-Victoria Idland, Bjørn Erik Neerland, Heidi Sandvig, Anniken Brugård, Johan Raeder, Frede Frihagen, Torgeir Bruun Wyller, Bjørnar Hassel, Jens Bollerslev, Leiv Otto Watne

**Affiliations:** 1 Oslo Delirium Research Group, Department of Geriatric Medicine, Oslo University Hospital, 0424 Oslo, Norway; 2 Department of Geriatric Medicine, Institute of Clinical Medicine, University of Oslo, 0424 Oslo, Norway; 3 Section of Specialized Endocrinology, Department of Endocrinology, Oslo University Hospital, 0424 Oslo, Norway; 4 Department Microbiology, Oslo University Hospital, 0424 Oslo, Norway; 5 Medical Department, Kristiansund Hospital, Møre og Romsdal Hospital Trust, 6508 Kristiansund, Norway; 6 Department of Anesthesiology, Oslo University Hospital, 0424 Oslo, Norway; 7 Division of Orthopedic Surgery, Oslo University Hospital, 0424 Oslo, Norway; 8 Department of Neurohabilitation, Oslo University Hospital, 0424 Oslo, Norway

**Keywords:** delirium, dementia, catecholamine, CSF biomarkers, hip fracture

## Abstract

Dopamine and noradrenaline are functionally connected to delirium and have been targets for pharmacological interventions but the biochemical evidence to support this notion is limited. To study the CSF levels of dopamine, noradrenaline and the third catecholamine adrenaline in delirium and dementia, these were quantified in three patient cohorts: (i) cognitively normal elderly patients (*n* = 122); (ii) hip fracture patients with or without delirium and dementia (*n* = 118); and (iii) patients with delirium precipitated by another medical condition (medical delirium, *n* = 26). Delirium was assessed by the Confusion Assessment Method. The hip fracture cohort had higher CSF levels of noradrenaline and adrenaline than the two other cohorts (both *P* < 0.001). Within the hip fracture cohort those with delirium (*n* = 65) had lower CSF adrenaline and dopamine levels than those without delirium (*n* = 52, *P* = 0.03, *P* = 0.002). Similarly, the medical delirium patients had lower CSF dopamine levels than the cognitively normal elderly (*P* < 0.001). Age did not correlate with the CSF catecholamine levels. These findings with lower CSF dopamine levels in hip fracture- and medical delirium patients challenge the theory of dopamine excess in delirium and question use of antipsychotics in delirium. The use of alpha-2 agonists with the potential to reduce noradrenaline release needs further examination.

## Teaser

Delirium, or ‘acute confusion’, is a common complication of acute illness in the elderly, for which there is no established treatment. Disturbance in three signalling substances, referred to as catecholamines, is suspected in delirium. Here, Henjum et al. studied CSF catecholamine levels and report alterations relevant for delirium treatment.

## Graphical Abstract

**Graphical Abstract:**
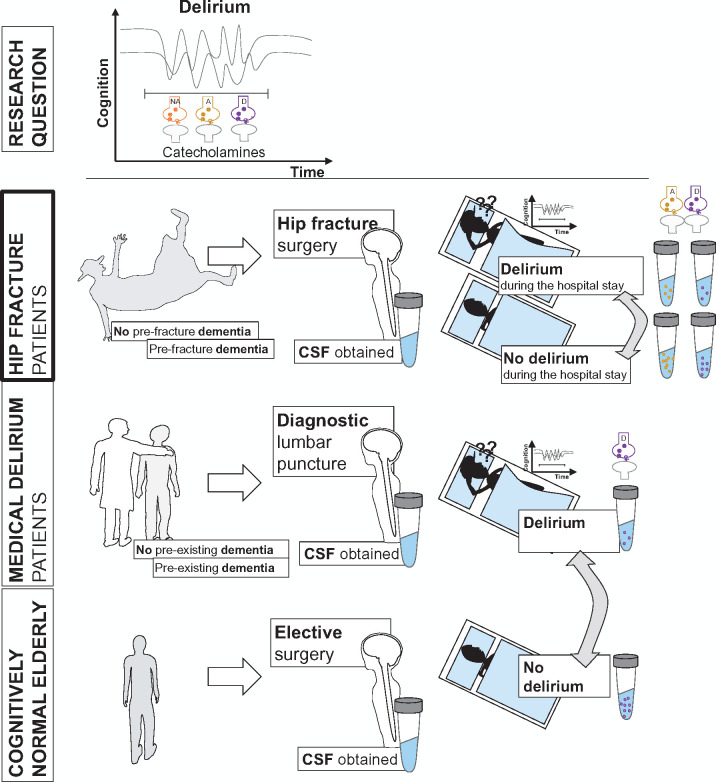

## Introduction

Acute, temporary disturbances in attention, awareness and cognition characterize delirium.^1^ This stressful syndrome, typically precipitated by acute illness in aged and demented people,^2^ is associated with prolonged hospitalization^3^ and subsequent cognitive decline.^4^^,^^5^ There are no established treatments but antipsychotics and α2-adrenergic-agonists are used.^6–8^

Delirium pathophysiology appears heterogeneous with multiple systems at play^9^ and may be influenced by dementia.^10–12^ Immune activation relates to neural activity^13^ and has received attention as an early mediator^12^^,^^14^^,^^15^ but neurotransmitter disturbances may result in the clinical presentation.^16^ The catecholamines noradrenaline and dopamine are involved in cognition,^17^ and are afflicted in dementias.^18^ Moreover, dopaminergic dysfunction is associated with hallucinations,^19^ a common delirium symptom. Noradrenaline additionally relates to key aspects of delirium as attention, arousal, sleep–wake, stress, pain and immune activation.^20^^,^^21^ Brain adrenergic neurons locate to a few brainstem areas that are activated by stress.^22^ Catecholamine activity is therefore believed excessive in delirium. Higher CSF levels of their precursors^10^ and the dopamine metabolite homovanillic acid (HVA)^23^ support this but the biochemical evidence is limited.

We asked if the CSF catecholamine levels are altered in delirium. To answer this study question CSF catecholamine levels were analysed (i) in hip fracture patients with and without delirium and dementia; (ii) in a second delirium group with delirium precipitated by another medical condition, and (iii) in cognitively normal elderly patients as a reference.

## Materials and methods

### Study participants

#### Hip fracture cohort

Hip fracture patients were recruited from the Oslo Orthogeriatric Trial (OOT) that included patients admitted to Oslo University Hospital from 2009 to 2012. The OOT was a randomized controlled trial evaluating the effect of an orthogeriatric service on delirium-associated long-term cognitive decline. All CSF samples available from this trial were included in the current study. During the hospital stay, delirium was assessed daily and until the fifth post-operative day by the Confusion Assessment Method.^24^ Based on all available clinical information, pre-fracture dementia status was determined by consensus in an expert panel. Dementia was diagnosed according to the International Classification of Diseases (ICD)-10 criteria^25^ without further information on dementia aetiology. For further details, see the previous publications.^26^^,^^27^

**Figure 1:**
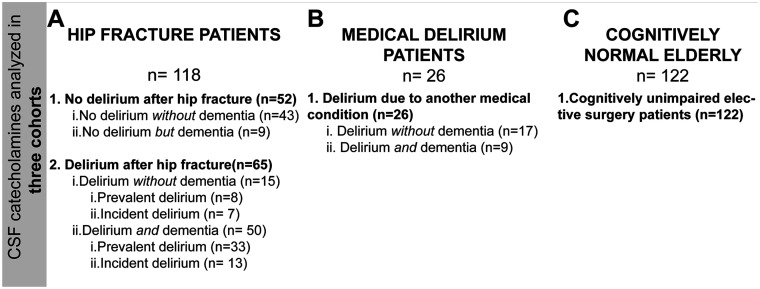
CSF catecholamine levels were analysed in three cohorts. (**A**) The hip fracture cohort included patients with and without dementia and delirium. Information about delirium at any time was missing for one patient and delirium status when CSF was obtained was missing for four patients. These patients were excluded from analyses requiring this information. (**B**) The medical delirium cohort included patients all with delirium due to another medical condition while (**C**) the final cohort included cognitively normal elderly patients.

The patients (*n* = 118) were grouped according to the presence of delirium at any time during the hospital stay (yes/no) and secondarily according to delirium status when CSF was sampled (prevalent; ongoing delirium at the time of CSF sampling, incident; free from delirium at the time of CSF sampling, but developed delirium after) and to pre-fracture dementia status (Fig. 1).

In sensitivity analyses, we excluded patients receiving medications directly related to the transmitter systems of interest, for noradrenaline and adrenaline relevant antidepressants (ATC-code N06A excluding N06A B Selective serotonin reuptake inhibitors; *n* = 13), for dopamine; antiparkinsonian agents (N04B; *n* = 2), antipsychotics (N05A *n* = 17, including preoperative haloperidole) and relevant antidepressants (N06A, non-selective monoamine reuptake inhibitors or N06A X16,18 and 21 serotonin-noradrenaline-reuptake inhibitors (*n* = 3), in total 20 patients due to medication overlap). No patients were prescribed MAO-A inhibitors or bupropion (N06A G and X12).

#### Medical delirium cohort

The medical patients were recruited from a prospective study at the same hospital from 2014 to 2015^12^^,^^28^ Briefly, these patients underwent lumbar puncture (LP) due to suspicion of an acute CNS infection. Patients in whom a CNS infection was ruled out and who were considered to have delirium triggered by another medical condition (most often pneumonia or urinary tract infection) were included in the current study (*n* = 26). Delirium was assessed either by the study physician by the Confusion Assessment Method (CAM), or by clinical evaluation of the treating physician. Dementia status was set from the hospital records. These patients formed a separate delirium group labelled ‘medical delirium’.

#### Cognitively normal elderly patients

A group of cognitively unimpaired (normal) elderly were recruited in 2012 and 2013 from patients scheduled for orthopaedic, urological or gynaecological elective surgery in spinal anaesthesia at Oslo University Hospital or Diakonhjemmet Hospital.^29^ In brief, the patients included turned 65 or more years the year of inclusion and had a Mini Mental State Examination (MMSE) score ≥28 at baseline. Those with signs of dementia within the first 5 years after inclusion were excluded (as detected in yearly cognitive assessments). Patients with previous stroke, Parkinson’s disease or other neurodegenerative disease likely affecting cognition at baseline were also excluded. Finally, 122 patients with sufficient CSF volume for catecholamine analyses were included.

**Standard protocol approvals, registrations and patient consents.** The study was performed in accordance with the Declaration of Helsinki. Informed consent was obtained from the patient or the closest relative if the patient was unable to give consent. The study was approved by the Regional Committee for Ethics in Medical and health research in Norway (REK 2009/450, REK 2011/2578 and REK 2011/2052).

#### CSF sampling and storage

For the hip fracture and the elective surgery patients, CSF was collected in connection with the surgery at the onset of spinal anaesthesia before administration of the anaesthetic agents. For the medical delirium patients, CSF was obtained in conjunction with the diagnostic LP at a median of one day after CNS symptom development. Hip fracture surgeries were performed at all hours (see Supplementary Fig. 1). Elective surgeries were performed at daytime (0800–1700) and all the cognitively normal elderly patients except one (surgery started 2100) underwent surgery within this timeframe. The medical delirium patients also underwent diagnostic LP at all hours, most at daytime (01:30–03:30: 2 patients, 09–12: 5 patients, 13:30–16: 13 patients, 18–24: 6 patients see also Supplementary Fig. 2). CSF was collected in polypropylene tubes, centrifuged and supernatant aliquots stored in polypropylene tubes at −80°C.^28–30^ The CSF samples were subject to one previous freeze–thaw cycle for the same analysis for all samples.

### CSF catecholamine analyses

The CSF concentrations of noradrenaline, adrenaline and dopamine were analysed in a single batch for each variable by means of an isocratic high-performance liquid chromatography (HPLC; Agilent Technologies, Santa Clara, CA, USA) system with a reversed-phase C-18 column (Chromsystem GmbH, Am Haag, Germany) and an electrochemical detection (ECD; Antec, Leyden Decade II SCC, Zoeterwoude, The Netherlands). The ECD had a three-electrode configuration system with a glassy carbon flow cell, an (*in situ* Ag/AgCl) reference electrode and an auxiliary electrode. The working potential between the working electrode and the auxiliary electrode was set to +0.60 V, range at 50 nA and sensitivity 10 nA. The HPLC conditions were Agilent analog/digital converter (ADC unit nA) units/volt 50.000 and peak width 0.133 min (data rate 2 Hz). Mobile phase flow; 1.0 ml/min, injection interval 15 min, the compressibility 100 × 10^−6^/bar, column temperature 40.0°C, injection volume 10.0 µl and run time 12 min. Mobile phase, calibration standard and internal standard reagents came from the same company, Chromsystem GmbH (order no. 5000, all CSF samples were measured with reagents from lot no 0318).

For quantitative results, all series started with a single point calibration sample (catecholamine calibration standard) as informed by the suppliers (Chromsystem GmbH and Antec, Scientific) for quantification of plasma catecholamines. This was here applied as CSF and plasma have comparable matrixes in HPLC-ECD analyses.^31^ An internal standard, 3,4-dihydroxybenzylamine (DHBA), was added to all CSF samples.

CSF samples were directly injected into the HPLC-ECD system (no CSF pre-preparation with solid-phase extraction- or sample clean-up columns) and run after the calibration standards. Peak for each component was integrated and identified from calibration retention time and the peak heights related to a concentration from the same standard.

A quality control (a CSF control pool) was injected for every tenth injection. The inter-assay coefficients of variation (CV; based on measurements of pooled CSF samples repeated in each assayed series), were 4.7%, 6.2% and 8.3% for noradrenaline, adrenaline and dopamine, respectively.

### Statistical analyses

The data were not normally distributed as judged by visual inspection and tests for normality (Shapiro–Wilk and Kolmogorow–Smirnov). Non-parametric analyses were, therefore, applied with central tendency and spread reported by median and the interquartile range (IQR). Group differences were analysed by Mann–Whitney or Kruskal–Wallis tests, and correlations by Spearman’s correlation coefficient (Spearman’s Rho; *R*_S_). The significance level was set at 0.05 and reported *P*-values are two-tailed.

Statistical analyses were performed by use of the Statistical Package for Social Sciences (SPSS, v.25; IBM, Armonk, NY, USA). Graphical illustrations were created with GraphPad Prism (v.8.0.3 Graph Pad Software, La Jolla, CA, USA).

### Data availability

The data that support the findings of this study are available from the corresponding author upon reasonable request.

## Results

### Catecholamine interrelations and relations to age, gender and diurnal rhythm

Noradrenaline, adrenaline and dopamine were detectable in all CSF samples in all three cohorts. CSF noradrenaline and adrenaline correlated positively among all patients (*R*_S_ = 0.58 *P* < 0.001, *n* = 266) and similarly in the three individual cohorts. CSF dopamine correlated with noradrenaline and adrenaline only in the hip fracture cohort (*R*_S_ = 0.24 and 0.29 with *P* = 0.01 and 0.001, *n* = 118).

The hip fracture patients were older than the cognitively normal elderly and the medical delirium patients (H(2) = 89.7, *P* < 0.001, *n* = 266, Table 1). However, neither noradrenaline, adrenaline nor dopamine correlated with age in the cognitively normal elderly (*R*_S_: 0.05, −0.04 and −0.07 respectively all *P*-values > 0.5, *n* = 122). The CSF catecholamine levels did not differ between the genders in the hip fracture cohort (U = 1357, 1400, 1384 all *P* > 0.75, *n* = 118). Cognitively normal elderly females had higher CSF dopamine levels than males (2.9 (1.9–3.5) versus 2.3 (1.7–3.0), U = 1437, *P* = 0.03, *n* = 60, *n* = 62)), but there were no differences in the CSF levels of noradrenaline and adrenaline (U = 1825 and 1524, *P* = 0.86, *P* = 0.09, *n* = 122). Acute hip fracture surgery and diagnostic LPs were performed at all hours but the CSF catecholamine levels did not differ in samples collected at different time intervals during the day (0:01–06:00, 06:01–12:00, 12:01–18:00, 18:01–24:00 Supplementary Figs. 1 and 2). Age, and time of surgery (hip fracture patients), were therefore not included in further analyses, while gender was accounted for in relevant analyses.

**Table 1:** Background characteristics of the three cohorts according to delirium status

	1. Cognitively normal	2. Medical delirium	Hip fracture			*P*			
			3. All	4. No delirium	5. Delirium	Group	Group	Group	Group
						1–3	2 vs 5	4 vs 5	1 vs 2
*N*	*122*	*26*	*118*	*52*	*65*				
Age	71.0 (68–76)	67.5 (61–77)	85 (80–89)	84 (72–88)	85 (81–90)	<0.001	<0.001	0.04	0.04
Gender									
Male	62 (50.8)	16 (61.5)	33 (28.0)	12 (23.1)	21 (32.3)				
Female	60 (49.2)	10 (38.5)	85 (72.0)	40 (72.9)	44 (67.7)				
Delirium									
No	122 (100)	0 (0)	52 (44.4)	52 (100)	0 (0)				
Yes	0 (0)	100 (100)	65 (55.6)	0 (0)	65 (100)				
Dementia									
No	122 (100)	17 (65.4)	58 (49.6)	43 (82.6)	15 (23.1)				
Yes	0 (0)	9 (34.6)	60 (50.4)	9 (17.3)	50 (76.9)				
CSF catechols									
Noradrenaline	13.4 (9.8–20.1)	17.8 (8.5–24.5)	38.2 (24.6–53.3)	39.4 (28.4–51.7)	35.4 (21.8–53.5)	<0.001	<0.001	0.21	0.30
Adrenaline	5.9 (3.9–8.3)	6.0 (3.6–7.4)	8.4 (5.3–12.7)	10.4 (6.7–13.6)	7.9 (4.5–12.0)	<0.001	0.049	0.03	0.96
Dopamine	2.6 (1.8–3.2)	1.2 (0.8–2.0)	1.5 (0.8–2.2)	2.0 (1.2–2.8)	1.3 (0.6–1.8)	<0.001	0.49	0.002	<0.001

Data are presented as number with percentages in brackets for gender, delirium and dementia. Age (years) and CSF catecholamine levels (nM) are presented as median and interquartile ranges as shown by the 25 and 75 percentiles. Two-tailed *P*-values are obtained by Kruskal–Wallis test for comparisons across the cohorts while Mann–Whitney U-test is applied for delirium group comparisons.

### CSF catecholamine levels in the three cohorts

**Figure 2:**
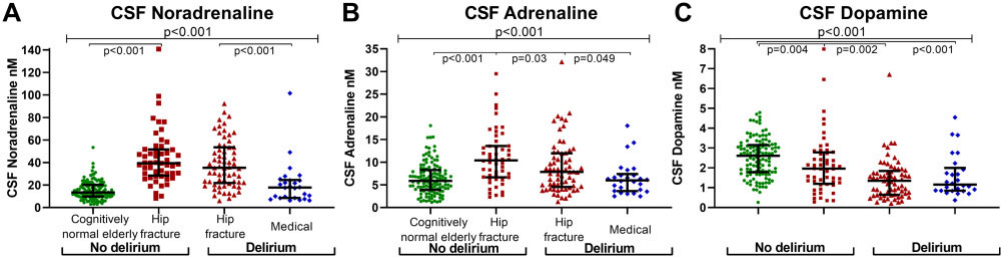
CSF catecholamine levels in patients with and without delirium in all cohorts. (**A**) CSF noradrenaline levels and (**B**) CSF adrenaline levels were higher among the hip fracture patients (all, *n* = 118) while (**C**) CSF dopamine levels were highest among the cognitively normal elderly (*n* = 122). Furthermore, hip fracture patients with delirium (*n* = 65) had compared those with no delirium (*n* = 52) (**B**) lower CSF adrenaline levels and (**C**) lower CSF dopamine levels. Medical delirium patients (*n*=26) also had lower CSF dopamine relative to the cognitively normal elderly. Larger and smaller lines represent median and interquartile range, respectively. Two-tailed *P*-values for comparison of two groups are obtained by Mann–Whitney U-test. Differences in the three cohorts (upper line) were analysed by Kruskal–Wallis test.

The cognitively normal elderly, medical delirium and hip fracture patients presented with distinct differences in the CSF catecholamine levels. While the hip fracture patients had the highest CSF levels of noradrenaline and adrenaline, CSF dopamine levels were highest among the cognitively normal elderly (H(2) = 111.9, 26.0 and 46.5 respectively all *P* < 0.001, *n* = 266, Fig. 2 and Table 1). CSF dopamine remained higher in the cognitively normal elderly when females and males were analysed separately (H(2) 37.73, *P* < 0.001, *n* = 155 and H(2) = 12.09, *P* = 0.002, *n* = 111).

### CSF catecholamine levels in hip fracture patients with and without delirium

As the higher levels of the adrenergic transmitters in the hip fracture patients could relate to the hip fracture further analyses of delirium and dementia among these patients were performed within this cohort. About half of the hip fracture patients experienced delirium during the hospital stay. These had lower levels of CSF adrenaline and dopamine than those without delirium (U = 1297, *P* = 0.03 and U = 1129.5, *P* = 0.002, respectively, *n* = 65 versus *n* = 52, Fig. 2 and Table 1).

**Figure 3:**
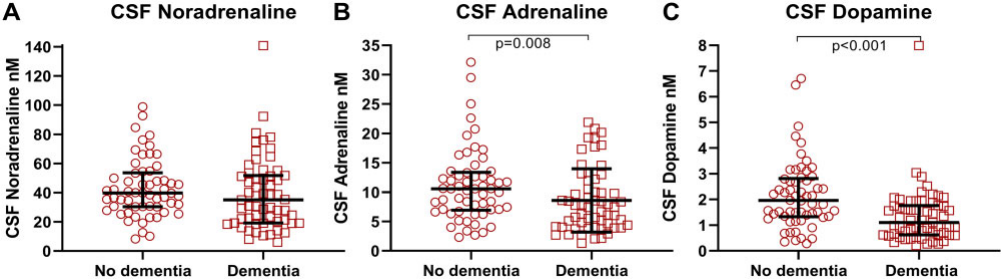
CSF catecholamine levels in hip fracture patients with and without pre-fracture dementia. (A) Hip fracture patients with pre-fracture dementia (*n* = 60) did compared to those with no pre-fracture dementia (*n* = 58) not have statistically significant lower CSF noradrenaline levels (*P* = 0.17) but had significantly lower CSF levels of (**B**) adrenaline and (**C**) dopamine. Larger and smaller lines represent median and interquartile range respectively. Two-tailed *P*-values are obtained by Mann–Whitney U-test.

Patients with pre-fracture dementia had lower CSF levels of adrenaline and dopamine than patients without pre-fracture dementia (U = 1251, *P* = 0.008 and U = 993.5, *P* < 0.001, *n* = 60 versus *n* = 58, Fig. 3 and Table 2). This remained significant for CSF dopamine but not for CSF adrenaline (*P* = 0.07) after exclusion of patients on relevant medications (see methods). The hip fracture patients were, therefore, divided according to pre-fracture dementia status for further analyses.

**Table 2:** Background characteristics; hip fracture patients with and without dementia

No pre-fracture dementia					
	All	No Delirium	Delirium		
			All	Incident	Prevalent
*N*	*58*	*43*	*15*	*7*	*8*
Age	84 (78–88)	84 (72–88)	85 (81–88)	86 (81–92)	85 (80–88)
Sex					
Male	17	11	6	3	3
Female	41	32	9	4	5
Time to surgery^a^	23 (16–34)	23 (14–31)	27 (21–36)	22 (13–32)	30 (22–37)
APACHE score^b^	8 (7–10)	8 (7–10)	9 (6–12)	8 (6–15)	10 (7–12)
CSF					
Noradrenaline	39.7 (30.4–53.7)	38.5 (27.8–49.9)	41.4 (34.6–66.4)	42.5 (40.9–72.4)	35.5 (25.2–51.5)
Adrenaline	10.6 (6.9–13.4)	10.6 (7.1–13.6)	10.6 (6.2–12.3)	12.0 (5.0–19.7)	8.6 (6.4–10.9)
Dopamine	2.0 (1.3–2.8)	2.0 (1.3–2.8)	1.8 (1.4–3.2)	2.3 (1.5–3.3)	1.6 (0.6–2.6)

**Pre-fracture dementia**					

	**All**	**No Delirium**	**Delirium**		
			**All**	**Incident**	**Prevalent**

*N*	*60*	*9*	*50*	*13*	*33*
Age	86 (81–90)	86 (71–92)	85 (81–90)	87 (84–91)	85 (80–88)
Sex					
Male	16	1	15	5	8
Female	44	8	35	8	25
Time to surgery^a^	26 (13–43)	28 (16–34)	26 (13–44)	18 (10–28)	38 (22–46)
APACHE score^b^	9 (8–10)	7 (6–9)	9 (8–10)	8 (7–9)	9 (8–11)
CSF					
Noradrenaline	35.2 (19.5–52.9)	47.1 (32.9–63.9)	30.3 (19.1–51.8)	30.8 (19.2–53.4)	30.6 (18.7–51.6)
Adrenaline	7.4 (4.2–11.6)	9.8 (4.8–15.4)	7.4 (4.1–10.5)	7.9 (4.7–12.9)	6.3 (4.0–11.0)
Dopamine	1.1 (0.6–1.8)	2.0 (0.7–2.5)	1.0 (0.6–1.7)	0.6 (0.5–2.1)	1.1 (0.7–1.6)

Data are presented as median and interquartiles. CSF noradrenaline, adrenaline and dopamine are given in nM, while age and time to surgery in years and hours, respectively. Information about delirium status was missing for one patient and delirium status when CSF was obtained was missing for four patients.

a Time to surgery; hours from hospital admission to surgery.

b APACHE score without blood-gas.

**Figure 4:**
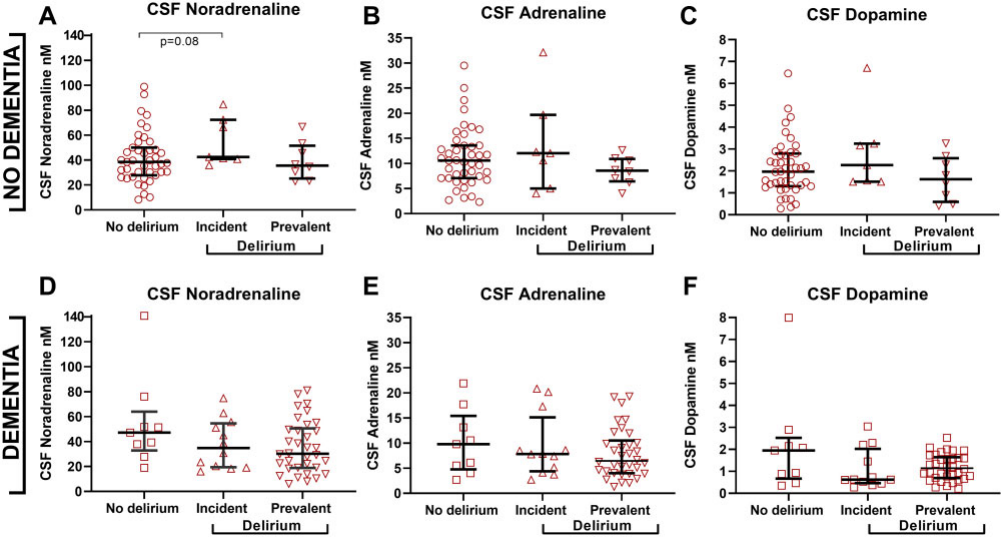
CSF catecholamine levels in delirium sub-grouped according to pre-fracture dementia status. (**A–C**) Among patients without pre-fracture dementia (**A**) CSF noradrenaline was although not significant higher in incident delirium (*n* = 7) compared to those with no delirium (*n* = 43; *P* = 0.08). (**D**–**F**) There were no significant differences among pre-fracture dementia patients. Larger and smaller lines represent median and interquartile range respectively. Two-tailed *P*-values are obtained by Mann–Whitney U-test.

The majority of the hip fracture patients with delirium had delirium superimposed on dementia (∼75%), while most patients without pre-fracture dementia did not develop delirium (∼75%, Table 2). When delirium was analysed in these patients separately, there were no differences in the CSF levels of noradrenaline, adrenaline and dopamine in patients with pre-fracture dementia (U = 151, *P* = 0.12, U = 185, *P* = 0.40 and U = 165, *P* = 0.21, *n* = 9 versus *n* = 50 Fig. 4 and Table 2). Likewise, there were no differences in patients with and without delirium among those without pre-fracture dementia (U = 271, 297 and 315 all *P* > 0.35, *n* = 43 versus *n* = 15 Fig. 4 and Table 2). However, further separation of patients with delirium before or delirium after surgery when CSF was obtained (prevalent and incident delirium) showed differences between patients with and without pre-fracture dementia. Patients without pre-fracture dementia but incident delirium had higher CSF noradrenaline levels than those with no delirium although this did not reach the significance level (U = 88, *P* = 0.08, *n* = 43 versus *n* = 7, Fig. 4 and Table 2). This was not observed for the CSF adrenaline and dopamine levels (U = 132, *P* = 0.62 and U = 108 *P* = 0.25, respectively, *n* = 43 versus *n* = 7). In the pre-fracture dementia strata, the medians were lower in both the incident and prevalent delirium groups but not significantly different (Fig. 4). Excluding patients receiving medication targeting noradrenaline and dopamine for the respective analyses did not alter the results.

### CSF catecholamine levels in medical delirium patients

Medical delirium patients had lower CSF dopamine levels than the cognitively normal elderly (U = 725, *P* < 0.001, *n* = 122 versus *n* = 26, Fig. 2 and Table 1). This remained significant when excluding medical delirium patients with pre-existing dementia from the analysis (U = 407, *P* < 0.001, *n* = 122, *n* = 17) and when analysing males and females separately (data not shown). The medical delirium patients and cognitively normal elderly had similar CSF noradrenaline and adrenaline levels (U = 1379.5, *P* = 0.30 and U = 1575, *P* = 0.96, *n* = 122 versus *n* = 26, Fig. 2 and Table 1).

CSF dopamine levels were similar in patients with delirium precipitated by a hip fracture and another medical condition (U = 765, *P* = 0.48, *n* = 65 versus *n* = 26, Fig. 2 and Table 1). In contrast, the hip fracture patients with delirium had higher CSF levels of noradrenaline and adrenaline than the medical delirium patients (U = 360, *P* < 0.001 and U = 621, *P* = 0.049, *n* = 65 versus *n* = 26, Fig. 2 and Table 1).

## Discussion

We examined central catecholamine activity by the CSF levels in delirium with or without pre-existing dementia in hip fracture patients, medical delirium patients and cognitively normal elderly. All three catecholamines were detected and quantified in CSF from all patients. The three cohorts presented with distinctly different catecholamine levels that seemed influenced by delirium and dementia.

The hip fracture patients differed from the other two cohorts with higher CSF levels of the adrenergic transmitters, in particular noradrenaline, consistent with activation of these systems by pain and stress.^22^^,^^32^ Although the other two cohorts had lower levels, the adrenergic transmitters correlated to the same degree in all patient groups. A few brainstem clusters, C1–C3, hold the required enzymes for adrenaline synthesis.^22^ Upon activation by stress these further activate noradrenergic clusters, including the locus coeruleus (LC) providing the main noradrenergic output in the brain.^21^ The role of adrenaline as a co-transmitter to glutamate is however unsettled^33^^,^^34^ but the observed correlations support a functional coupling between the adrenergic transmitters.

Contrasting the anticipation of excessive dopaminergic activity in delirium^9^ hip fracture patients with delirium had lower CSF dopamine levels than those with no delirium and the medical delirium patients had lower CSF dopamine levels than the cognitively normal elders. Antipsychotics antagonizing dopaminergic transmission, yet with different receptor affinities, have been evaluated as delirium therapeutics.^6^ Haloperidol has been the preferred agent,^35^ but trial outcomes have been inconsistent. Two recent larger studies do, however, report lack of effect on delirium treatment^36^ and prevention.^37^ Meta-analyses including atypical antipsychotics also do not support their use in delirium.^38^^,^^39^ The lower CSF levels of dopamine in delirium in our study align with the negative outcomes of antipsychotics. However, antipsychotics have complex receptor profiles and their lack of efficacy may be attributed to interference with other systems. A recent study found higher homovanillic acid-levels in delirium rather pointing to increased dopaminergic activity in neurologic patients.^23^ These delirium patients were however fairly young (mean age ∼38 years) and represent a rarer delirium population with autoimmune limbic encephalitis or other (co-)morbidities as epilepsy and HIV-infections in which the pathophysiology may differ from the typical, aged delirium patients.

The hip fracture patients with dementia, the majority with delirium superimposed on dementia, had lower CSF levels of adrenaline and dopamine. Lower CSF dopamine align with dopamine reductions in dementia in post-mortem studies^40^ and lower CSF homovanillic acid levels in Alzheimer’s Disease^41^ as diagnosed by the NINCDS-ADRA criteria. Although the locus coeruleus is affected in dementias,^42^ there was no difference in the CSF noradrenaline levels in patients with and without pre-fracture dementia. This may be due to activation of compensatory mechanisms as dendritic and axonal sprouting or increases in tyrosine hydroxylase^43^ increasing noradrenaline release from the remaining neurons. Another reasoning may be that the hip fracture influences the CSF noradrenaline levels to such an extent that differences due to existing pathology were masked. Already in the preclinical phase do Alzheimer’s disease seem to pose an increased risk of delirium.^29^^,^^44^ The mechanisms underlying delirium may be different in people with dementia and should be addressed in future studies.

Activated in arousal, attention and at wake, noradrenaline clinically relates to delirium. Dexmedetomidine is an α2-agonist with analgesic, sedative and anxiolytic effects attributed to stimulation of α2-autoreceptors in the locus coeruleus and spinal cord, mediating negative feedback thus reducing noradrenaline release.^45^^,^^46^ In ventilated patients sedation with dexmedetomidine is associated with reduced delirium occurrence compared to other sedatives^47^^,^^48^ and more ventilator free days compared to placebo.^49^ Beneficial effects are also seen in non-ventilated patients^50^ although with some controversy.^51^ CSF noradrenaline was higher in incident delirium patients without pre-fracture dementia but did not reach the significance level. This should be investigated in future studies as it suggests an increase in noradrenergic activity in the phase before delirium is clinically evident supporting dexmedetomidine as a delirium preventive agent.^52^ The medical delirium patients were all admitted to the hospital with ongoing delirium and CSF changes in the phase prior to clinically evident delirium could not be explored in these patients. However, a recent study found serum noradrenaline levels associated with medical ICU-acquired delirium^53^ suggesting that noradrenaline in delirium may be systemically elevated.

A strength of this study is the large sample size allowing for subgrouping of patients based on delirium status at the time of CSF sampling and pre-fracture dementia status. Still, although a large study in the context of delirium, the number of individuals in some subgroup analyses was limited. This may increase the likelihood of false negative but also false positive results.^54^ All available samples collected in a previous study (the OOT) were included, a priori power calculations were not performed. As an explorative study, the results should be confirmed in larger follow-up studies.^55^ The inclusion of two additional reference groups, one with and one without delirium, helped understanding the interplay between delirium and the hip fracture itself upon CSF catecholamine levels. It also allowed us to see similarities between separate delirium precipitators. The cross-sectional design is a limitation of the study but longitudinal CSF delirium studies are challenging to perform.

The retrospective classification of dementia without further information on dementia aetiology is a limitation of the study. People with Dementia with Lewy bodies may have higher CSF noradrenaline levels than other dementias and a higher risk of delirium.^56^^,^^57^ However, few patients would be expected to have Dementia with Lewy bodies as Alzheimer’s disease dementia with mixed pathology is the most common cause of dementia in aged populations.^58^^,^^59^ Poor sleep could also influence the study but could not be adjusted for which is another study limitation.

## Conclusions

Catecholamine activity was assessed by the CSF noradrenaline, adrenaline and dopamine levels. Hip fracture and medical delirium patients presented with lower CSF dopamine levels but dementia and delirium alterations overlapped in the hip fracture patients. In patients without pre-fracture dementia CSF noradrenaline was higher in incident delirium although not statistically significant. These findings should be replicated in larger studies but are in line with restricting the use of antipsychotics for delirium patients and further exploring use of α2-agonists.

## Supplementary material

Supplementary material is available at *Brain Communications* online.

## Supplementary Material

fcab121_Supplementary_Data

## Abbreviations

CAM =: confusion assessment method;
HPLC-ECD =: high-performance liquid chromatography with electrochemical detection;
HVA =: homovanillic acid;
LC =: locus coeruleus;
LP =: lumbar puncture

## References

[1] American Psychiatric Assocation. Diagnostic and statistical manual of mental disorders, 5th ed. Washinghton DC: American Psychiatric Publishing; 2013.

[2] Ahmed S, Leurent B, Sampson EL. Risk factors for incident delirium among older people in acute hospital medical units: A systematic review and meta-analysis. Age Ageing. 2014;43(3):326–333. 24610863 10.1093/ageing/afu022 PMC4001175

[3] Robinson TN, Raeburn CD, Tran ZV, Angles EM, Brenner LA, Moss M. Postoperative delirium in the elderly: Risk factors and outcomes. Ann Surg. 2009;249(1):173–178. 19106695 10.1097/SLA.0b013e31818e4776

[4] Krogseth M, Wyller TB, Engedal K, Juliebo V. Delirium is an important predictor of incident dementia among elderly hip fracture patients. Dement Geriatr Cogn Disord. 2011;31(1):63–70. 21212674 10.1159/000322591

[5] Davis DH, Muniz Terrera G, Keage H, et al Delirium is a strong risk factor for dementia in the oldest-old: A population-based cohort study. Brain. 2012;135(Pt 9):2809–2816. 22879644 10.1093/brain/aws190 PMC3437024

[6] Oh ES, Fong TG, Hshieh TT, Inouye SK. Delirium in older persons: Advances in diagnosis and treatment. JAMA. 2017;318(12):1161–1174. 28973626 10.1001/jama.2017.12067 PMC5717753

[7] Hov KR, Neerland BE, Undseth Ø, et al The Oslo Study of Clonidine in Elderly Patients with Delirium; LUCID: A randomised placebo-controlled trial. Int J Geriatr Psychiatry. 2019;34(7):974–981. 30901487 10.1002/gps.5098

[8] Reznik ME, Slooter AJC. Delirium management in the ICU. Curr Treat Options Neurol. 2019;21(11):59- 31724092 10.1007/s11940-019-0599-5

[9] Maldonado JR. Delirium pathophysiology: An updated hypothesis of the etiology of acute brain failure. Int J Geriatr Psychiatry. 2018;33(11):1428–1457. 29278283 10.1002/gps.4823

[10] Watne LO, Idland AV, Fekkes D, et al Increased CSF levels of aromatic amino acids in hip fracture patients with delirium suggests higher monoaminergic activity. BMC Geriatr. 2016;16:149- 27484129 10.1186/s12877-016-0324-0 PMC4970288

[11] Hov KR, Bolstad N, Idland AV, et al Cerebrospinal fluid S100B and Alzheimer's disease biomarkers in hip fracture patients with delirium. Dement Geriatr Cogn Dis Extra. 2017;7(3):374–385. 29282410 10.1159/000481853 PMC5731172

[12] Henjum K, Quist-Paulsen E, Zetterberg H, Blennow K, Nilsson LNG, Watne LO. CSF sTREM2 in delirium-relation to Alzheimer's disease CSF biomarkers Abeta42, t-tau and p-tau. J Neuroinflammation. 2018;15(1):304- 30390679 10.1186/s12974-018-1331-1 PMC6215363

[13] Kettenmann H, Hanisch UK, Noda M, Verkhratsky A. Physiology of microglia. Physiol Rev. 2011;91(2):461–553. 21527731 10.1152/physrev.00011.2010

[14] Cerejeira J, Lagarto L, Mukaetova-Ladinska EB. The immunology of delirium. Neuroimmunomodulation. 2014;21(2-3):72–78. 24557038 10.1159/000356526

[15] Hall RJ, Watne LO, Idland AV, et al Cerebrospinal fluid levels of neopterin are elevated in delirium after hip fracture. J Neuroinflammation. 2016;13(1):170- 27357281 10.1186/s12974-016-0636-1 PMC4928278

[16] Klein RS, Garber C, Howard N. Infectious immunity in the central nervous system and brain function. Nat Immunol. 2017;18(2):132–141. 28092376 10.1038/ni.3656 PMC5815515

[17] Robbins TW, Arnsten AF. The neuropsychopharmacology of fronto-executive function: Monoaminergic modulation. Annu Rev Neurosci. 2009;32:267–287. 19555290 10.1146/annurev.neuro.051508.135535 PMC2863127

[18] Trillo L, Das D, Hsieh W, et al Ascending monoaminergic systems alterations in Alzheimer's disease. translating basic science into clinical care. Neurosci Biobehav Rev. 2013;37(8):1363–1379. 23707776 10.1016/j.neubiorev.2013.05.008

[19] Lauretani F, Ceda GP, Maggio M, Nardelli A, Saccavini M, Ferrucci L. Capturing side-effect of medication to identify persons at risk of delirium. Aging Clin Exp Res. 2010;22(5-6):456–458. 21422797 10.1007/bf03324944 PMC6109713

[20] Maclullich AM, Ferguson KJ, Miller T, de Rooij SE, Cunningham C. Unravelling the pathophysiology of delirium: A focus on the role of aberrant stress responses. J Psychosom Res. 2008;65(3):229–238. 18707945 10.1016/j.jpsychores.2008.05.019 PMC4311661

[21] Szabadi E. Functional neuroanatomy of the central noradrenergic system. J Psychopharmacol. 2013;27(8):659–693. 23761387 10.1177/0269881113490326

[22] Guyenet PG, Stornetta RL, Bochorishvili G, Depuy SD, Burke PG, Abbott SB. C1 neurons: The body's EMTs. Am J Physiol Regul Integr Comp Physiol. 2013;305(3):R187–204. 23697799 10.1152/ajpregu.00054.2013 PMC3743001

[23] Ramirez-Bermudez J, Perez-Neri I, Montes S, et al Dopaminergic hyperactivity in neurological patients with delirium. Arch Med Res. 2019;50(8):477–483. 32018069 10.1016/j.arcmed.2019.11.002

[24] Inouye SK, van Dyck CH, Alessi CA, Balkin S, Siegal AP, Horwitz RI. Clarifying confusion: The confusion assessment method. A new method for detection of delirium. Ann Intern Med. 1990;113(12):941–948. 2240918 10.7326/0003-4819-113-12-941

[25] World Health Organization. The ICD-10 Classification of Mental and Behavioural Disorders Diagnostic criteria for research. World Health Organization. 1993. Available at http://www.who.int/classifications/icd/en/GRNBOOK.pdf. Accessed 26 March 2021.

[26] Watne LO, Torbergsen AC, Conroy S, et al The effect of a pre- and postoperative orthogeriatric service on cognitive function in patients with hip fracture: Randomized controlled trial (Oslo Orthogeriatric Trial). BMC Med. 2014;12:63. 24735588 10.1186/1741-7015-12-63 PMC4022270

[27] Wyller TB, Watne LO, Torbergsen A, et al The effect of a pre- and post-operative orthogeriatric service on cognitive function in patients with hip fracture. The protocol of the Oslo Orthogeriatrics Trial. BMC Geriatr. 2012;12:36. 22817102 10.1186/1471-2318-12-36 PMC3583172

[28] Quist-Paulsen E, Aukrust P, Kran AB, et al High neopterin and IP-10 levels in cerebrospinal fluid are associated with neurotoxic tryptophan metabolites in acute central nervous system infections. J Neuroinflammation. 2018;15(1):327. 30470234 10.1186/s12974-018-1366-3 PMC6260858

[29] Idland AV, Wyller TB, Stoen R, et al Preclinical amyloid-beta and axonal degeneration pathology in delirium. J Alzheimers Dis. 2016;55(1):371–379. 10.3233/JAD-160461 27662296

[30] Watne LO, Hall RJ, Molden E, et al Anticholinergic activity in cerebrospinal fluid and serum in individuals with hip fracture with and without delirium. J Am Geriatr Soc. 2014;62(1):94–102. 24383557 10.1111/jgs.12612

[31] Hooshfar S, Basiri B, Bartlett MG. Development of a surrogate matrix for cerebral spinal fluid for liquid chromatography/mass spectrometry based analytical methods. Rapid Commun Mass Spectrom. 2016;30(7):854–858. 26969926 10.1002/rcm.7509

[32] Valentino RJ, Van Bockstaele E. Convergent regulation of locus coeruleus activity as an adaptive response to stress. Eur J Pharmacol. 2008;583(2-3):194–203. 18255055 10.1016/j.ejphar.2007.11.062 PMC2349983

[33] Abbott SB, Kanbar R, Bochorishvili G, Coates MB, Stornetta RL, Guyenet PG. C1 neurons excite locus coeruleus and A5 noradrenergic neurons along with sympathetic outflow in rats. J Physiol. 2012;590(12):2897–2915. 22526887 10.1113/jphysiol.2012.232157 PMC3448155

[34] Holloway BB, Stornetta RL, Bochorishvili G, Erisir A, Viar KE, Guyenet PG. Monosynaptic glutamatergic activation of locus coeruleus and other lower brainstem noradrenergic neurons by the C1 cells in mice. J Neurosci. 2013;33(48):18792–18805. 24285886 10.1523/JNEUROSCI.2916-13.2013 PMC3841449

[35] Patel RP, Gambrell M, Speroff T, et al Delirium and sedation in the intensive care unit: Survey of behaviors and attitudes of 1384 healthcare professionals. Crit Care Med. 2009;37(3):825–832. 19237884 10.1097/CCM.0b013e31819b8608 PMC3719180

[36] van den Boogaard M, Slooter AJC, Bruggemann RJM, et al REDUCE Study Investigators. Effect of haloperidol on survival among critically ill adults with a high risk of delirium: The REDUCE randomized clinical trial. JAMA. 2018;319(7):680–690. 29466591 10.1001/jama.2018.0160 PMC5839284

[37] Girard TD, Exline MC, Carson SS, et al; MIND-USA Investigators. Haloperidol and ziprasidone for treatment of delirium in critical illness. N Engl J Med. 2018;379(26):2506–2516. 30346242 10.1056/NEJMoa1808217 PMC6364999

[38] Nikooie R, Neufeld KJ, Oh ES, et al Antipsychotics for treating delirium in hospitalized adults: A systematic review. Ann Intern Med. 2019;171(7):485–495. 31476770 10.7326/M19-1860

[39] Oh ES, Needham DM, Nikooie R, et al Antipsychotics for preventing delirium in hospitalized adults: A systematic review. Ann Intern Med. 2019;171(7):474–484. 31476766 10.7326/M19-1859

[40] Adolfsson R, Gottfries CG, Roos BE, Winblad B. Changes in the brain catecholamines in patients with dementia of Alzheimer type. Br J Psychiatry. 1979;135:216–223. 486847 10.1192/bjp.135.3.216

[41] Blennow K, Wallin A. Clinical heterogeneity of probable Alzheimer's disease. J Geriatr Psychiatry Neurol. 1992;5(2):106–113. 1590911 10.1177/002383099200500208

[42] Zarow C, Lyness SA, Mortimer JA, Chui HC. Neuronal loss is greater in the locus coeruleus than nucleus basalis and substantia nigra in Alzheimer and Parkinson diseases. Arch Neurol. 2003;60(3):337–341. 12633144 10.1001/archneur.60.3.337

[43] Szot P, White SS, Greenup JL, Leverenz JB, Peskind ER, Raskind MA. Compensatory changes in the noradrenergic nervous system in the locus ceruleus and hippocampus of postmortem subjects with Alzheimer's disease and dementia with Lewy bodies. J Neurosci. 2006;26(2):467–478. 16407544 10.1523/JNEUROSCI.4265-05.2006 PMC6674412

[44] Cunningham EL, McGuinness B, McAuley DF, et al CSF beta-amyloid 1-42 concentration predicts delirium following elective arthroplasty surgery in an observational cohort study. Ann Surg. 2019;269(6):1200–1205. 31082921 10.1097/SLA.0000000000002684

[45] Khan ZP, Ferguson CN, Jones RM. alpha-2 and imidazoline receptor agonists. Their pharmacology and therapeutic role. Anaesthesia. 1999;54(2):146–165. 10215710 10.1046/j.1365-2044.1999.00659.x

[46] Starke K. Presynaptic autoreceptors in the third decade: Focus on alpha2-adrenoceptors. J Neurochem. 2001;78(4):685–693. 11520889 10.1046/j.1471-4159.2001.00484.x

[47] Pandharipande PP, Pun BT, Herr DL, et al Effect of sedation with dexmedetomidine vs lorazepam on acute brain dysfunction in mechanically ventilated patients: The MENDS randomized controlled trial. JAMA. 2007;298(22):2644–2653. 18073360 10.1001/jama.298.22.2644

[48] Maldonado JR, Wysong A, van der Starre PJ, Block T, Miller C, Reitz BA. Dexmedetomidine and the reduction of postoperative delirium after cardiac surgery. Psychosomatics. 2009;50(3):206–217. 19567759 10.1176/appi.psy.50.3.206

[49] Reade MC, Eastwood GM, Bellomo R, et al; for the DahLIA Investigators and the Australian and New Zealand Intensive Care Society Clinical Trials Group. Effect of dexmedetomidine added to standard care on ventilator-free time in patients with agitated delirium: A randomized clinical trial. JAMA. 2016;315(14):1460–1468. 26975647 10.1001/jama.2016.2707

[50] Skrobik Y, Duprey MS, Hill NS, Devlin JW. Low-dose nocturnal dexmedetomidine prevents ICU delirium. A randomized, placebo-controlled trial. Am J Respir Crit Care Med. 2018;197(9):1147–1156. 29498534 10.1164/rccm.201710-1995OC

[51] Deiner S, Luo X, Lin HM, et al and the Dexlirium Writing Group. Intraoperative infusion of dexmedetomidine for prevention of postoperative delirium and cognitive dysfunction in elderly patients undergoing major elective noncardiac surgery: A randomized clinical trial. JAMA Surg. 2017;152(8):e171505. 28593326 10.1001/jamasurg.2017.1505 PMC5831461

[52] Su X, Meng ZT, Wu XH, et al Dexmedetomidine for prevention of delirium in elderly patients after non-cardiac surgery: A randomised, double-blind, placebo-controlled trial. Lancet. 2016;388(10054):1893–1902. 27542303 10.1016/S0140-6736(16)30580-3

[53] Yasuda Y, Nishikimi M, Nishida K, et al Relationship between serum norepinephrine levels at ICU admission and the risk of ICU-acquired delirium: Secondary analysis of the melatonin evaluation of lowered inflammation of ICU trial. Crit Care Explor. 2020;2(2):e0082. 32211614 10.1097/CCE.0000000000000082 PMC7069595

[54] Button KS, Ioannidis JP, Mokrysz C, et al Power failure: Why small sample size undermines the reliability of neuroscience. Nat Rev Neurosci. 2013;14(5):365–376. 23571845 10.1038/nrn3475

[55] Althouse AD. Adjust for multiple comparisons? It's not that simple. Ann Thorac Surg. 2016;101(5):1644–1645. 27106412 10.1016/j.athoracsur.2015.11.024

[56] Vardy E, Holt R, Gerhard A, Richardson A, Snowden J, Neary D. History of a suspected delirium is more common in dementia with Lewy bodies than Alzheimer's disease: A retrospective study. Int J Geriatr Psychiatry. 2014;29(2):178–181. 23722989 10.1002/gps.3986

[57] Janssens J, Vermeiren Y, Fransen E, et al Cerebrospinal fluid and serum MHPG improve Alzheimer's disease versus dementia with Lewy bodies differential diagnosis. Alzheimers Dement (Amsterdam, Netherlands). 2018;10:172–181. 10.1016/j.dadm.2018.01.002 PMC5852321 29552632

[58] Barker WW, Luis CA, Kashuba A, et al Relative frequencies of Alzheimer disease, Lewy body, vascular and frontotemporal dementia, and hippocampal sclerosis in the State of Florida Brain Bank. Alzheimer Dis Assoc Disord. 2002;16(4):203–212. 12468894 10.1097/00002093-200210000-00001

[59] Jellinger KA, Attems J. Prevalence of dementia disorders in the oldest-old: An autopsy study. Acta Neuropathol. 2010;119(4):421–433. 20204386 10.1007/s00401-010-0654-5

