# Supplementary material for: Cerebrospinal fluid catecholamines in delirium and dementia
Source: Brain Commun. 2021 May 29;3(3):fcab121. doi: 10.1093/braincomms/fcab121 (PMC8374970; doi:10.1093/braincomms/fcab121)
Supplement: fcab121_Supplementary_Data [file fcab121_Supplementary_Data.zip › Revision 1.pdf]

OXFORD UNIVERSITY PRESS | Brain Communications

Cerebrospinal fluid catecholamines in delirium and dementia

Journal:	*Brain Communications*
Manuscript ID	BRAINCOM-2020-227.R1
Manuscript Type:	Original Article
Date Submitted by the Author:	15-Dec-2020
Complete List of Authors:	Henjum, Kristi; University of Oslo, Department of Geriatric Medicine<br>Godang, Kristin ; Oslo University Hospital, Dept. of Endocrinology<br>Quist-Paulsen, Else; Oslo University Hospital, Department Microbiology<br>Idland, Ane-Victoria; Oslo University Hospital, Oslo Delirium Research Group, Department of Geriatric Medicine<br>Neerland, Bjørn Erik; Oslo University Hospital, Oslo Delirium Research Group, Department of Geriatric Medicine<br>Sandvig, Heidi; Møre og Romsdal Hospital Trust, Medical Department<br>Brugård, Anniken; Oslo University Hospital, Oslo Delirium Research Group, Deparment of Geriatric Medicine<br>Ræder, Johan; University of Oslo, Institute of Clinical Medicine<br>Frihagen, Frede; Oslo University Hospital, Dept. of Orthopaedic Surgery<br>Wyller, Torgeir; University of Oslo, Institute of Clinical Medicine; Oslo University Hospital, Dept. of Geriatric Medicine<br>Hassel, Bjørnar; Oslo University Hospital, Department of Neurohabilitation<br>Bollerslev, Jens; Oslo University Hospital, Department of Endocrinology<br>Watne, Leiv; University of Oslo, Institute of Clinical Medicine; Oslo University Hospital, Dept. of Geriatric Medicine
Keywords:	Delirium, Dementia, Catecholamine, CSF biomarkers, Hip fracture

SCHOLARONE™
Manuscripts

Title: Cerebrospinal fluid catecholamines in delirium and dementia

Running title: Catecholamines in delirium and dementia

Kristi Henjum PhD[1,2*], Kristin Godang BSc[3], Else Quist-Paulsen MD-PhD[4], Ane-Victoria Idland MD[1], Bjørn Erik Neerland MD-PhD[1], Heidi Sandvig MD[5,], Anniken Brugård[1], Johan Raeder MD-PhD[6], Frede Frihagen MD-PhD[7], Torgeir Bruun Wyller MD-PhD[1,2], Bjørnar Hassel MD-PhD [8], Jens Bollerslev MD-PhD [2,3] and Leiv Otto Watne MD-PhD [1]

1 Oslo Delirium Research Group, Department of Geriatric Medicine, Oslo University Hospital, Oslo, Norway
2 Department of Geriatric Medicine, Institute of Clinical Medicine, University of Oslo, Oslo Norway
3 Section of Specialized Endocrinology, Dept. of Endocrinology, Oslo University Hospital, Oslo, Norway.
4 Department Microbiology, Oslo University Hospital, Oslo, Norway.
5 Medical department, Kristiansund Hospital, Møre og Romsdal Hospital Trust, Kristiansund Norway
6 Department of Anesthesiology, Oslo University Hospital, Oslo, Norway
7 Division of Orthopedic Surgery, Oslo University Hospital, Oslo, Norway
8 Department of Neurohabilitation, Oslo University Hospital, Oslo, Norway

*Corresponding author:
Kristi Henjum, Department of Geriatric Medicine, Institute of Clinical Medicine, University of Oslo, PO 4956 Nydalen, 0424 OSLO, Norway. E-mail kristi.henjum@medisin.uio.no

For Review Only

ABSTRACT

Dopamine and noradrenaline are functionally connected to delirium and have been targets for pharmacological interventions but the biochemical evidence to support this notion is limited. To study the cerebrospinal fluid (CSF) levels of dopamine, noradrenaline and the third catecholamine adrenaline in delirium and dementia these were quantified in three patient cohorts: 1) Cognitively normal elderly patients (n=122) 2) Hip fracture patients with or without delirium and dementia (n=118) 3) Patients with delirium precipitated by another medical condition (medical delirium, n=26). Delirium was assessed by the Confusion Assessment Method. The hip fracture cohort had higher CSF levels of noradrenaline and adrenaline than the two other cohorts (both $p<0.001$). Within the hip fracture cohort those with delirium (n=65) had lower CSF adrenaline and dopamine levels than those without delirium (n=52, p=0.03, p=0.002). Similarly the medical delirium patients had lower CSF dopamine levels than the cognitively normal elderly ($p<0.001$). Age did not correlate with the CSF catecholamine levels. These findings with lower CSF dopamine levels in hip fracture- and medical delirium patients challenge the theory of dopamine excess in delirium and question use of antipsychotics in delirium. Use of alpha-2 agonists with the potential to reduce noradrenaline release needs further examination.

Keywords: Delirium, dementia, catecholamine, CSF biomarkers, hip fracture

Abbreviations: CAM: Confusion Assessment Method, HPLC-ECD: High-performance liquid chromatography with electrochemical detection, HVA: Homovanillic acid, LC: Locus coeruleus, LP: Lumbar puncture.

For Review Only

INTRODUCTION

Acute, temporary disturbances in attention, awareness and cognition characterize delirium (American Psychiatric Assocation, 2013). This stressful syndrome, typically precipitated by acute illness in aged and demented people (Ahmed *et al.*, 2014), is associated with prolonged hospitalization (Robinson *et al.*, 2009) and subsequent cognitive decline (Krogseth *et al.*, 2011; Davis *et al.*, 2012). There are no established treatments but antipsychotics and α2-adrenergic-agonists are used (Oh *et al.*, 2017; Hov *et al.*, 2019; Reznik and Slooter, 2019).

Delirium pathophysiology appears heterogeneous with multiple systems at play (Maldonado, 2018) and may be influenced by dementia (Watne *et al.*, 2016; Hov *et al.*, 2017; Henjum *et al.*, 2018). Immune activation relates to neural activity (Kettenmann *et al.*, 2011) and has received attention as an early mediator (Cerejeira *et al.*, 2014; Hall *et al.*, 2016; Henjum *et al.*, 2018) but neurotransmitter disturbances may result in the clinical presentation (Klein *et al.*, 2017). The catecholamines noradrenaline and dopamine are involved in cognition (Robbins and Arnsten, 2009), and are afflicted in dementias (Trillo *et al.*, 2013). Moreover dopaminergic dysfunction is associated with hallucinations (Lauretani *et al.*, 2010) a common delirium symptom. Noradrenaline additionally relates to key aspects of delirium as attention, arousal, sleep-wake, stress, pain and immune activation (Maclullich *et al.*, 2008; Szabadi, 2013). Brain adrenergic neurons locates to a few brainstem areas that are activated by stress (Guyenet *et al.*, 2013). Catecholamine activity is therefore believed excessive in delirium. Higher cerebrospinal fluid (CSF) levels of their precursors (Watne *et al.*, 2016) and the dopamine metabolite homovanillic acid (HVA) (Ramirez-Bermudez *et al.*, 2019) supports this but the biochemical evidence is limited.

For Review Only

We asked if the CSF catecholamine levels are altered in delirium. To answer this study question CSF catecholamine levels were analyzed in 1) hip fracture patients with and without delirium and dementia, 2) in a second delirium group with ~~group with~~ delirium precipitated by another medical condition ~~as a second delirium group~~ and in 3) cognitively normal elderly patients as a reference ~~to answer the study question~~.

MATERIALS AND METHODS

Study participants

Hip fracture cohort

Hip fracture patients were recruited from the Oslo Orthogeriatric Trial (OOT) that included patients admitted to Oslo University Hospital from 2009 to 2012 (Wyller *et al.*, 2012; Watne *et al.*, 2014b). In brief, during the hospital stay delirium was assessed daily and until the fifth post-operative day by the Confusion Assessment Method (CAM) (Inouye *et al.*, 1990). Based on all available clinical information ~~P~~pre-fracture dementia status was determined by consensus in an expert panel. Dementia was diagnosed according to the International Classification of Diseases (ICD)-10 criteria (World Health Organization, 1993) without further information on dementia etiology ~~based on all available clinical information~~.

The patients (n=118) were grouped according to presence of delirium at any time during the hospital stay (yes/no) and secondarily according to delirium status when CSF was sampled (prevalent; ongoing delirium at the time of CSF sampling, incident; free from delirium at the time of CSF sampling, but developed delirium after) and to pre-fracture dementia status (Fig. 1).

In sensitivity analyses, we excluded patients receiving medications directly related to the transmitter systems of interest, for noradrenaline and adrenaline relevant antidepressants (ATC-code N06A excluding N06A B Selective serotonin reuptake inhibitors; n=13), for dopamine; antiparkinsonian agents (N04B; n=2), antipsychotics (N05A n= 17, including preoperative haloperidole), and relevant antidepressants (N06A, non-selective monoamine reuptake inhibitors or N06A X16,18 and 21 serotonin-noradrenaline-reuptake inhibitors (n= 3), in total 20 patients due to medication overlap. No patients were prescribed MAO-A inhibitors or bupropion (N06A G and X12).

Medical delirium cohort

The medical patients were recruited from a prospective study at the same hospital from 2014 to 2015 (Henjum *et al.*, 2018; Quist-Paulsen *et al.*, 2018). Briefly, these patients underwent lumbar puncture (LP) due to suspicion of an acute central nervous system (CNS) infection. Patients in whom a CNS infection was ruled out and who were considered to have delirium triggered by another medical condition (most often pneumonia or urinary tract infection) were included in the current study (n=26). Delirium was assessed either by the study physician with CAM, or by clinical

For Review Only

evaluation of the treating physician. Dementia status was set from the hospital records. These patients formed a separate delirium group labeled "medical delirium".

Cognitively normal elderly patients

A group of cognitively unimpaired (normal) elderly were recruited in 2012 and 2013 from patients scheduled for orthopedic, urological or gynecological elective surgery in spinal anesthesia at Oslo University Hospital or Diakonhjemmet Hospital (Idland *et al.*, 2017). In brief, the patients included turned 65 or more years the year of inclusion and had a Mini Mental State Examination (MMSE) score ≥28 at baseline. Those with signs of dementia within the first 5 years after inclusion were excluded (as detected in yearly cognitive assessments). Patients with previous stroke, Parkinson's disease or other neurodegenerative disease likely affecting cognition at baseline were also excluded. Finally, 122 patients with sufficient CSF volume for catecholamine analyses were included.

Standard Protocol Approvals, Registrations, and Patient Consents

The study was performed in accordance with the Declaration of Helsinki. Informed consent was obtained from the patient or the closest relative if the patient was unable to give consent. The study was approved by the Regional Committee for Ethics in Medical and health research in Norway (REK 2009/450, REK 2011/2578 and REK 2011/2052).

CSF sampling and storage

For the hip fracture and the elective surgery patients, CSF was collected in connection with the surgery at onset of spinal anesthesia before administration of the anesthetic agents. For the medical delirium patients CSF was obtained in conjunction with the diagnostic LP at a median of one day after CNS symptom development. ~~,~~ Hip fracture surgeries were performed at all hours (Supplementary Fig.S1). Elective surgeries were performed at daytime only and CSF from such patients were therefore all sampled at daytime. Diagnostic LP of the medical delirium patients was also performed at all hours but for most at daytime (01:30-03: 30: 2 patients, 09-12; 5 patients; 13:30-16: 13 patients 18-24; 6 patients, see also Supplementary Fig. S2)~~for most patients (n= 19) between 09-18~~. CSF was collected in polypropylene tubes, centrifuged and supernatant aliquots stored in polypropylene tubes at -80^{0}C (Watne *et al.*, 2014a; Idland *et al.*, 2017; Quist-Paulsen *et*

For Review Only

al., 2018). The CSF samples were subject to one previous freeze-thaw cycle for the same analysis for all samples.

CSF catecholamine analyses

The CSF concentrations of noradrenaline, adrenaline and dopamine were analyzed in a single batch for each variable by means of an isocratic high-performance liquid chromatography (HPLC; Agilent Technologies, Santa Clara, CA, USA) system with a reversed-phase C-18 column (Chromsystem GmbH, Am Haag, Germany) and an electrochemical detection (ECD; Antec, Leyden Decade II SCC, Zoeterwoude, The Netherlands). The ECD had a three electrode configuration system with a glassy carbon flow cell, an (in situ Ag/AgCl) reference electrode and an auxiliary electrode. The working potential between the working electrode and the auxiliary electrode was set to +0.60V, range at 50nA and sensitivity 10nA. tThe HPLC conditions were sat to: Agilent analog/digital converter (ADC unit nA) units/volt 50.000 and peak width 0.133min (data rate 2Hz). Mobile phase flow; 1.0ml/min, injection interval 15min, the compressibility 100x10^{-6}/bar, column temperature 40.0 °C, injection volume 10.0µl and run time 12min. Mobile phase, calibration standard and internal standard reagents came from the same company, Chromsystem GmbH.

For quantitative results all series started with a single point calibration sample (catecholamine calibration standard). An internal standard, 3,4-dihydroxybenzylamine (DHBA) was added to all CSF samples.

CSF samples were directly injected in the HPLC-ECD system (no CSF pre-preparation with solid-phase extraction- or sample clean up columns) and run after the calibration standards. Peak for each component were integrated and identified from calibration retention time and the peak heights related to a concentration from the same standard. Single point calibration with a regression line through origo is shown appropriate for plasma catecholamines by the reagent supplier Chromsystem GmbH. CSF and plasma have comparable matrixes in HPLC-ECD analyzes (Hooshfar *et al.*, 2016) and single point calibration was applied for the current CSF analyses.

Quality controls (a CSF control pool) was injected for every tenth injection. The inter-assay coefficients of variation (CV; based on measurements of pooled CSF samples repeated in each

For Review Only

assayed series), were 4.7%, 6.2% and 8.3% for noradrenaline, adrenaline and dopamine, respectively.

Statistical analyses

The data were not normally distributed as judged by visual inspection and tests for normality (Shapiro-Wilk and Kolmogorow-Smirnov). Non-parametric analyses were therefore applied with central tendency and spread reported by median and the interquartile range (IQR). Group differences were analyzed by Mann-Whitney- or Kruskal-Wallis tests, and correlations by Spearman's correlation coefficient (Spearman's Rho; R_S). The significance level was set at 0.05 and reported p-values two-tailed.

Statistical analyses were performed by use of the Statistical Package for Social Sciences (SPSS, v.25; IBM, Armonk, NY, USA). Graphical illustrations were created with GraphPad Prism (v.8.0.3 Graph Pad Software, La Jolla, CA, USA)

For Review Only

Data availability

The data that support the findings of this study are available from the corresponding author upon reasonable request.

RESULTS

Catecholamine interrelations and relations to age, gender and diurnal rhythm.

Noradrenaline, adrenaline and dopamine were detectable in all CSF samples in all three cohorts. CSF noradrenaline and adrenaline correlated positively among all patients (R_S = 0.58 p<0.001, n=266) and similarly in the three individual cohorts. CSF dopamine correlated with noradrenaline and adrenaline only in the hip fracture cohort (R_S=0.24 and 0.29 with p=0.01 and 0.001, n=118).

The hip fracture patients were older than the cognitively normal elderly and the medical delirium patients (H(2)=89.7, p<0.001, n=266, Table 1). However neither noradrenaline, adrenaline nor dopamine correlated with age in the cognitively normal elderly (R_S: -–0.05, --0.054 and -0.07 respectively with all p-values>0.5, n=122). The CSF catecholamine levels did not differ between the genders in the hip fracture cohort (U= 1357, 1400, 1384 all p>0.75, n=118). Cognitively normal

elderly females had higher CSF dopamine levels than males (2.9 (1.9-3.5) vs 2.3 (1.7-3.0), U= 1437, p= 0.03, n=60, n=62) but there were no difference in the CSF levels of noradrenaline and adrenaline (U= 1825 and 1524, p= 0.86, p=0.09, n= 122). The Acute hip fracture patients surgery and diagnostic LPs were underwent acute surgery performed at all hours. The CSF catecholamines did not display diurnal rhythmicity neither in hip fracture nor in the medical delirium patients (Supplementary Fig.S1 and S2) but CSF noradrenaline did not display with diurnal rhythmicity (Supplementary Fig.S1). Age, and time of surgery (hip fracture patients), were therefore not included in further analyses while gender were accounted for in relevant analyses.

CSF catecholamine levels in the three cohorts

The cognitively normal elderly, medical delirium and hip fracture patients presented with distinct differences in the CSF catecholamine levels. While the hip fracture patients had the highest CSF levels of noradrenaline and adrenaline, CSF dopamine levels were highest in the cognitively normal elderly (H(2) =111.9, 26.0, 46.5 respectively all p<0.001, n=266, Fig. 2 and Table 1). CSF dopamine remained higher in the cognitively normal elderly when females and males were analyzed separately (H(2) 37.73, p<0.001, n=155 and H(2)= 12.09, p=0.002, n=111).

CSF catecholamine levels in hip fracture patients with and without delirium

As the higher levels of the adrenergic transmitters in the hip fracture patients could relate to the hip fracture further analyses of delirium and dementia these patients were performed within this cohort. About half of the hip fracture patients experienced delirium during the hospital stay. These had lower levels of CSF adrenaline and dopamine than those without delirium (U= 1297, p=0.03 and U= 1129.5, p=0.002 respectively, n=65 vs n=52, Fig 2. and Table 1).

Patients with pre-fracture dementia had compared to those without pre-fracture dementia, lower CSF levels of adrenaline and dopamine than patients without pre-fracture dementia (U=1251, p=0.008 and U=993.5, p<0.001, n=60 vs n=58, Table 2, Fig. 2). Fig. 3 and Table 2). This remained significant for CSF dopamine but not for CSF adrenaline (p=0.07) after exclusion of patients on relevant medications (see methods). The hip fracture patients were therefore divided according to pre-fracture dementia status for further analyses.

The majority of the hip fracture patients with delirium had delirium superimposed on dementia (~75%), while most patients without pre-fracture dementia did not develop delirium (~75%, Table 2). When delirium was analyzed in these patients separately, there were no differences in the CSF levels of noradrenaline, adrenaline and dopamine in patients with pre-fracture dementia (U= 151, p= 0.12, U= 185, p= 0.40 and U= 165, p= 0.21, n= 9 vs n=50 Fig. 3 and Table 2). Likewise there were no differences in patients with and without delirium among those without pre-fracture dementia (U = 271, 297 and 315 all p>0.35, n=43 vs n=15 Fig. 3 and Table 2). However further separation of patients with delirium before or delirium after surgery when CSF was obtained (prevalent and incident delirium) showed differences between patients with and without pre-fracture dementia. Patients without pre-fracture dementia but incident delirium had higher CSF noradrenaline than those with no delirium although this did not reach the significance level (U= 88, p= 0.08, n= 43 vs n= 7, Fig. 4 and Table 2). This was not observed for CSF adrenaline and dopamine (U=132, p=0.62 and U=108 p=0.25, respectively, n=43 vs n=7). In the pre-fracture dementia strata the medians were lower in both the incident and prevalent delirium groups but not significantly different (Fig. 4). Excluding patients receiving medication targeting noradrenaline and dopamine for the respective analyses did not alter the results.

CSF catecholamine levels in medical delirium patients

Medical delirium patients had lower CSF dopamine than the cognitively normal elderly (U=725, p<0.001, n= 122 vs n=26, Fig. 2 and Table 1). This remained significant when excluding medical delirium patients with pre-existing dementia from the analysis (U= 407, p<0.001, n=122, n=17) and when analyzing males and females separately (data not shown). The medical delirium patients and cognitively normal elderly had similar CSF noradrenaline and adrenaline levels (U=1379.5, p= 0.30 and U= 1575 p=0.96, n=122 vs n=26, Fig. 2 and Table 1).

CSF dopamine levels were similar in patients with delirium precipitated by a hip fracture and another medical condition (U=765, p=0.48, n=65 vs n=26, Fig. 2 and Table 1). In contrast the hip fracture patients with delirium had higher CSF levels of noradrenaline and adrenaline than the medical delirium patients (U= 360, p<0.001 and U= 621, p=0.049 n=65 vs n=26, Fig. 2 and Table 1).

DISCUSSION

For Review Only

We examined central catecholamine activity by the CSF levels in delirium with or without pre-existing dementia in hip fracture patients, medical delirium patients and cognitively normal elderly. All three catecholamines were detected and quantified in CSF from all patients. The three cohorts presented with distinctly different catecholamine levels that seemed influenced by delirium and dementia.

The hip fracture patients differed from the other two cohorts with higher CSF levels of the adrenergic transmitters, in particular noradrenaline, consistent with activation of these systems by pain and stress (Valentino and Van Bockstaele, 2008; Guyenet *et al.*, 2013). Although the other two cohorts had lower levels, the adrenergic transmitters correlated to the same degree in all patient groups. A few brainstem clusters, C1-C3, hold the required enzymes for adrenaline synthesis (Guyenet *et al.*, 2013). Upon activation by stress these further activate noradrenergic clusters, including the locus coeruleus (LC) providing the main noradrenergic output in the brain (Szabadi, 2013). The role of adrenaline as a co-transmitter to glutamate is however unsettled (Abbott *et al.*, 2012; Holloway *et al.*, 2013) but the observed correlations support a functional coupling between the adrenergic transmitters.

Contrasting the anticipation of excessive dopaminergic activity in delirium (Maldonado, 2018) hip fracture patients with delirium had lower CSF dopamine levels than those with no delirium and the medical delirium patients had lower CSF dopamine levels than the cognitively normal elders. Antipsychotics antagonizing dopaminergic transmission, yet with different receptor affinities, have been evaluated as delirium therapeutics (Oh *et al.*, 2017). Haloperidol has been the preferred agent (Patel *et al.*, 2009), but trial outcomes have been inconsistent. Two recent larger studies do however report lack of effect on delirium treatment (van den Boogaard *et al.*, 2018) and prevention (Girard *et al.*, 2018). Meta-analyses including atypical antipsychotics also do not support their use in delirium (Nikooie *et al.*, 2019; Oh *et al.*, 2019). The lower CSF levels of dopamine in delirium in our study align with the negative outcomes of antipsychotics. However, antipsychotics have complex receptor profiles and their lack of efficacy may be attributed to interference with other systems. A recent study found higher HVA-levels in delirium rather pointing to increased dopaminergic activity in neurologic patients (Ramirez-Bermudez *et al.*, 2019). These delirium patients were however fairly young (mean age ~38 years) and represent a rarer delirium population

For Review Only

with autoimmune limbic encephalitis or other (co-)morbidities as epilepsy and HIV-infections in which the pathophysiology may differ from the typical, aged delirium patients.

The hip fracture patients with dementia, the majority with delirium superimposed on dementia, had lower CSF levels of adrenaline and dopamine. Lower CSF dopamine align with dopamine reductions in dementia in post-mortem studies (Adolfsson *et al.*, 1979) and lower CSF HVA levels in Alzheimer's Disease (Blennow and Wallin, 1992) as diagnosed by the NINCDS-ADRA criteria. Although the LC is affected in dementias (Zarow *et al.*, 2003) there were no difference in the CSF noradrenaline levels in patients with and without pre-fracture dementia. This may be due to activation of compensatory mechanisms as dendritic and axonal sprouting or increases in tyrosine hydroxylase (Szot *et al.*, 2006) increasing noradrenaline release from the remaining neurons. Another reasoning may be that the hip fracture influence the CSF noradrenaline levels to such an extent that differences due to existing pathology were masked. Already in the preclinical phase do Alzheimer's disease seem to pose an increased risk of delirium (Idland *et al.*, 2017; Cunningham *et al.*, 2019)The mechanisms underlying delirium may be different in people with dementia and should be addressed in future studies.

Activated in arousal, attention and at wake, noradrenaline clinically relates to delirium. Dexmedetomidine is an α2-agonist with analgesic, sedative and anxiolytic effects attributed to stimulation of α2-autoreceptors in the LC and spinal cord, mediating negative feedback thus reducing noradrenaline release (Khan *et al.*, 1999; Starke, 2001). In ventilated patients sedation with dexmedetomidine is associated with reduced delirium occurrence compared to other sedatives (Pandharipande *et al.*, 2007; Maldonado *et al.*, 2009) and more ventilator free days compared to placebo (Reade *et al.*, 2016). Beneficial effects are also seen in non-ventilated patients (Skrobik *et al.*, 2018) although with some controversy (Deiner *et al.*, 2017). CSF noradrenaline was higher in incident delirium patients without pre-fracture dementia but did not reach the significance level. This should be investigated in future studies as it suggest an increase in noradrenergic activity in the phase before delirium is clinically evident supporting dexmedetomidine as a delirium preventive agent (Su *et al.*, 2016). The medical delirium patients were all admitted to the hospital with ongoing delirium and CSF changes in the phase prior to clinically evident delirium could not be explored in these patients.

https://mc.manuscriptcentral.com/braincom

A strength of this study is the large sample size allowing for subgrouping of patients based on delirium status at the time of CSF sampling and pre-fracture dementia status. Still, although a large study in the context of delirium, the study power for subgroup analyses was limited. This may increase the likelihood of false negative but also false positive results (Button *et al.*, 2013). As an explorative study the results should be confirmed in larger follow up studies (Althouse, 2016)The findings should therefore be pursued in larger studies. The inclusion of two additional reference groups, one with and one without delirium, helped understanding the interplay between delirium and the hip fracture itself upon on CSF catecholamine levels. It also allowed us to see similarities between separate delirium precipitators. The cross-sectional design is a limitation of the study but longitudinal CSF delirium studies is challenging to perform.

The retrospective classification of dementia without further information on dementia etiology is a limitation of the study. People with Dementia with Lewy bodies (DLB) may have higher CSF noradrenaline levels than other dementias and a higher risk of delirium (Vardy *et al.*, 2014; Janssens *et al.*, 2018). However few patients would be expected to have DLB as Alzheimer's disease dementia with mixed pathology as this is the most common cause of dementia in aged populations (Barker *et al.*, 2002; Jellinger and Attems, 2010). Poor sleep could also influence the study but could not be adjusted for which is another study limitation.

For Review Only

Conclusions

Catecholamine activity was assessed by the CSF noradrenaline, adrenaline and dopamine levels. Hip fracture and medical delirium patients presented with lower CSF dopamine levels but dementia and delirium alterations overlapped in the hip fracture patients. In patients without pre-fracture dementia CSF noradrenaline was higher in incident delirium although not statistical significant. These findings should be replicated in larger studies but are in line with restricting the use of antipsychotics for delirium patients and further exploring use of α2-agonists.

ACKNOWLEDGEMENTS

We would like to thank the patients and staff at the Orthopedic Department, the Gynecology Department, the Urology Department, Department of Internal Medicine and the Anesthesiology Department at Oslo University Hospital and the Surgery Department and the Anesthesiology

Department at Diakonhjemmet Hospital in Oslo. We also thank research nurses Elisabeth Fragaat and Tone Fredriksen for help in data collection. Grants from the Norwegian Health Association and the South-Eastern Norway Regional Health Authorities funded this work but the sponsors had no role in the design and study conduct nor approval of the manuscript.

FUNDING

This work was funded by grants from the Norwegian Health Association and the South-Eastern Norway Regional Health Authorities. The sponsors had no role in the design and conduct of the study, the collection, management, analysis, and interpretation of the data, or the preparation, review, and approval of the manuscript.

COMPETING INTERESTS

KH, KG, LOW, EQP, BEN, LOW, BH, FF, AI, HS, JR and JB report no competing interests. TBW has given a lecture for Bayer for which the Oslo Delirium Research Group received the speaker honoraria.

For Review Only

SUPPLEMENTARY MATERIAL See file supplementary material

REFERENCES

Abbott, S.B., Kanbar, R., Bochorishvili, G., Coates, M.B., Stornetta, R.L., Guyenet, P.G., 2012. C1 neurons excite locus coeruleus and A5 noradrenergic neurons along with sympathetic outflow in rats. J. Physiol. 590(12), 2897-2915.

Adolfsson, R., Gottfries, C.G., Roos, B.E., Winblad, B., 1979. Changes in the brain catecholamines in patients with dementia of Alzheimer type. Br. J. Psychiatry 135, 216-223.

Ahmed, S., Leurent, B., Sampson, E.L., 2014. Risk factors for incident delirium among older people in acute hospital medical units: a systematic review and meta-analysis. Age Ageing 43(3), 326-333.

Althouse, A.D., 2016. Adjust for Multiple Comparisons? It's Not That Simple. Ann. Thorac. Surg. 101(5), 1644-1645.

American Psychiatric Assocation, 2013. Diagnostic and Statistical Manual of Mental Disorders. 5th ed, 5th ed., American Psychiatric Publishing.

Barker, W.W., Luis, C.A., Kashuba, A., Luis, M., Harwood, D.G., Loewenstein, D., et al., 2002. Relative frequencies of Alzheimer disease, Lewy body, vascular and frontotemporal dementia, and hippocampal sclerosis in the State of Florida Brain Bank. Alzheimer Dis. Assoc. Disord. 16(4), 203-212.

Blennow, K., Wallin, A., 1992. Clinical heterogeneity of probable Alzheimer's disease. J. Geriatr. Psychiatry Neurol. 5(2), 106-113.

Button, K.S., Ioannidis, J.P., Mokrysz, C., Nosek, B.A., Flint, J., Robinson, E.S., et al., 2013. Power failure: why small sample size undermines the reliability of neuroscience. Nat. Rev. Neurosci. 14(5), 365-376.

Cerejeira, J., Lagarto, L., Mukaetova-Ladinska, E.B., 2014. The immunology of delirium. Neuroimmunomodulation 21(2-3), 72-78.

Cunningham, E.L., McGuinness, B., McAuley, D.F., Toombs, J., Mawhinney, T., O'Brien, S., et al., 2019. CSF Beta-amyloid 1-42 Concentration Predicts Delirium Following Elective Arthroplasty Surgery in an Observational Cohort Study. Ann. Surg. 269(6), 1200-1205.

Deiner, S., Luo, X., Lin, H.M., Sessler, D.I., Saager, L., Sieber, F.E., et al., 2017. Intraoperative Infusion of Dexmedetomidine for Prevention of Postoperative Delirium and Cognitive Dysfunction in Elderly Patients Undergoing Major Elective Noncardiac Surgery: A Randomized Clinical Trial. JAMA surgery 152(8), e171505.

Girard, T.D., Exline, M.C., Carson, S.S., Hough, C.L., Rock, P., Gong, M.N., et al., 2018. Haloperidol and Ziprasidone for Treatment of Delirium in Critical Illness. N. Engl. J. Med. 379(26), 2506-2516.

Guyenet, P.G., Stornetta, R.L., Bochorishvili, G., Depuy, S.D., Burke, P.G., Abbott, S.B., 2013. C1 neurons: the body's EMTs. Am. J. Physiol. Regul. Integr. Comp. Physiol. 305(3), R187-204.

Hall, R.J., Watne, L.O., Idland, A.V., Raeder, J., Frihagen, F., MacLullich, A.M., et al., 2016. Cerebrospinal fluid levels of neopterin are elevated in delirium after hip fracture. J. Neuroinflammation 13(1), 170.

Henjum, K., Quist-Paulsen, E., Zetterberg, H., Blennow, K., Nilsson, L.N.G., Watne, L.O., 2018. CSF sTREM2 in delirium-relation to Alzheimer's disease CSF biomarkers Abeta42, t-tau and p-tau. J. Neuroinflammation 15(1), 304.

Holloway, B.B., Stornetta, R.L., Bochorishvili, G., Erisir, A., Viar, K.E., Guyenet, P.G., 2013. Monosynaptic glutamatergic activation of locus coeruleus and other lower brainstem noradrenergic neurons by the C1 cells in mice. J. Neurosci. 33(48), 18792-18805.

Hooshfar, S., Basiri, B., Bartlett, M.G., 2016. Development of a surrogate matrix for cerebral spinal fluid for liquid chromatography/mass spectrometry based analytical methods. Rapid Commun. Mass Spectrom. 30(7), 854-858.

Hov, K.R., Bolstad, N., Idland, A.V., Zetterberg, H., Blennow, K., Chaudhry, F.A., et al., 2017. Cerebrospinal Fluid S100B and Alzheimer's Disease Biomarkers in Hip Fracture Patients with Delirium. Dement. Geriatr. Cogn. Dis. Extra 7(3), 374-385.

Idland, A.V., Wyller, T.B., Stoen, R., Eri, L.M., Frihagen, F., Raeder, J., et al., 2017. Preclinical Amyloid-beta and Axonal Degeneration Pathology in Delirium. J. Alzheimers Dis. 55(1), 371-379.

Inouye, S.K., van Dyck, C.H., Alessi, C.A., Balkin, S., Siegal, A.P., Horwitz, R.I., 1990. Clarifying confusion: the confusion assessment method. A new method for detection of delirium. Ann. Intern. Med. 113(12), 941-948.

Janssens, J., Vermeiren, Y., Fransen, E., Aerts, T., Van Dam, D., Engelborghs, S., et al., 2018. Cerebrospinal fluid and serum MHPG improve Alzheimer's disease versus dementia with Lewy bodies differential diagnosis. Alzheimer's & dementia (Amsterdam, Netherlands) 10, 172-181.

Jellinger, K.A., Attems, J., 2010. Prevalence of dementia disorders in the oldest-old: an autopsy study. Acta Neuropathol. 119(4), 421-433.

For Review Only

Kettenmann, H., Hanisch, U.K., Noda, M., Verkhratsky, A., 2011. Physiology of microglia. Physiol. Rev. 91(2), 461-553.

Khan, Z.P., Ferguson, C.N., Jones, R.M., 1999. alpha-2 and imidazoline receptor agonists. Their pharmacology and therapeutic role. Anaesthesia 54(2), 146-165.

Klein, R.S., Garber, C., Howard, N., 2017. Infectious immunity in the central nervous system and brain function. Nat. Immunol. 18(2), 132-141.

Lauretani, F., Ceda, G.P., Maggio, M., Nardelli, A., Saccavini, M., Ferrucci, L., 2010. Capturing side-effect of medication to identify persons at risk of delirium. Aging Clin. Exp. Res. 22(5-6), 456-458.

Maclullich, A.M., Ferguson, K.J., Miller, T., de Rooij, S.E., Cunningham, C., 2008. Unravelling the pathophysiology of delirium: a focus on the role of aberrant stress responses. J. Psychosom. Res. 65(3), 229-238.

Maldonado, J.R., 2018. Delirium pathophysiology: An updated hypothesis of the etiology of acute brain failure. Int. J. Geriatr. Psychiatry 33(11), 1428-1457.

Maldonado, J.R., Wysong, A., van der Starre, P.J., Block, T., Miller, C., Reitz, B.A., 2009. Dexmedetomidine and the reduction of postoperative delirium after cardiac surgery. Psychosomatics 50(3), 206-217.

Nikooie, R., Neufeld, K.J., Oh, E.S., Wilson, L.M., Zhang, A., Robinson, K.A., et al., 2019. Antipsychotics for Treating Delirium in Hospitalized Adults: A Systematic Review. Ann. Intern. Med.

Oh, E.S., Fong, T.G., Hshieh, T.T., Inouye, S.K., 2017. Delirium in Older Persons: Advances in Diagnosis and Treatment. JAMA 318(12), 1161-1174.

Oh, E.S., Needham, D.M., Nikooie, R., Wilson, L.M., Zhang, A., Robinson, K.A., et al., 2019. Antipsychotics for Preventing Delirium in Hospitalized Adults: A Systematic Review. Ann. Intern. Med.

Pandharipande, P.P., Pun, B.T., Herr, D.L., Maze, M., Girard, T.D., Miller, R.R., et al., 2007. Effect of sedation with dexmedetomidine vs lorazepam on acute brain dysfunction in mechanically ventilated patients: the MENDS randomized controlled trial. JAMA 298(22), 2644-2653.

Patel, R.P., Gambrell, M., Speroff, T., Scott, T.A., Pun, B.T., Okahashi, J., et al., 2009. Delirium and sedation in the intensive care unit: survey of behaviors and attitudes of 1384 healthcare professionals. Crit. Care Med. 37(3), 825-832.

Quist-Paulsen, E., Aukrust, P., Kran, A.B., Dunlop, O., Ormaasen, V., Stiksrud, B., et al., 2018. High neopterin and IP-10 levels in cerebrospinal fluid are associated with neurotoxic tryptophan metabolites in acute central nervous system infections. J. Neuroinflammation 15(1), 327.

Ramirez-Bermudez, J., Perez-Neri, I., Montes, S., Nente, F., Ramirez-Abascal, M., Carrillo-Mezo, R., et al., 2019. Dopaminergic Hyperactivity in Neurological Patients with Delirium. Arch. Med. Res. 50(8), 477-483.

Reade, M.C., Eastwood, G.M., Bellomo, R., Bailey, M., Bersten, A., Cheung, B., et al., 2016. Effect of Dexmedetomidine Added to Standard Care on Ventilator-Free Time in Patients With Agitated Delirium: A Randomized Clinical Trial. JAMA 315(14), 1460-1468.

Robbins, T.W., Arnsten, A.F., 2009. The neuropsychopharmacology of fronto-executive function: monoaminergic modulation. Annu. Rev. Neurosci. 32, 267-287.

Robinson, T.N., Raeburn, C.D., Tran, Z.V., Angles, E.M., Brenner, L.A., Moss, M., 2009. Postoperative delirium in the elderly: risk factors and outcomes. Ann. Surg. 249(1), 173-178.

For Review Only

Skrobik, Y., Duprey, M.S., Hill, N.S., Devlin, J.W., 2018. Low-Dose Nocturnal Dexmedetomidine Prevents ICU Delirium. A Randomized, Placebo-controlled Trial. Am. J. Respir. Crit. Care Med. 197(9), 1147-1156.

Starke, K., 2001. Presynaptic autoreceptors in the third decade: focus on alpha2-adrenoceptors. J. Neurochem. 78(4), 685-693.

Su, X., Meng, Z.T., Wu, X.H., Cui, F., Li, H.L., Wang, D.X., et al., 2016. Dexmedetomidine for prevention of delirium in elderly patients after non-cardiac surgery: a randomised, double-blind, placebo-controlled trial. Lancet 388(10054), 1893-1902.

Szabadi, E., 2013. Functional neuroanatomy of the central noradrenergic system. J Psychopharmacol 27(8), 659-693.

Szot, P., White, S.S., Greenup, J.L., Leverenz, J.B., Peskind, E.R., Raskind, M.A., 2006. Compensatory changes in the noradrenergic nervous system in the locus ceruleus and hippocampus of postmortem subjects with Alzheimer's disease and dementia with Lewy bodies. J. Neurosci. 26(2), 467-478.

Trillo, L., Das, D., Hsieh, W., Medina, B., Moghadam, S., Lin, B., et al., 2013. Ascending monoaminergic systems alterations in Alzheimer's disease. translating basic science into clinical care. Neurosci. Biobehav. Rev. 37(8), 1363-1379.

Valentino, R.J., Van Bockstaele, E., 2008. Convergent regulation of locus coeruleus activity as an adaptive response to stress. Eur. J. Pharmacol. 583(2-3), 194-203.

van den Boogaard, M., Slooter, A.J.C., Bruggemann, R.J.M., Schoonhoven, L., Beishuizen, A., Vermeijden, J.W., et al., 2018. Effect of Haloperidol on Survival Among Critically Ill Adults With a High Risk of Delirium: The REDUCE Randomized Clinical Trial. JAMA 319(7), 680-690.

Vardy, E., Holt, R., Gerhard, A., Richardson, A., Snowden, J., Neary, D., 2014. History of a suspected delirium is more common in dementia with Lewy bodies than Alzheimer's disease: a retrospective study. Int. J. Geriatr. Psychiatry 29(2), 178-181.

Watne, L.O., Hall, R.J., Molden, E., Raeder, J., Frihagen, F., MacLullich, A.M., et al., 2014a. Anticholinergic activity in cerebrospinal fluid and serum in individuals with hip fracture with and without delirium. J. Am. Geriatr. Soc. 62(1), 94-102.

Watne, L.O., Idland, A.V., Fekkes, D., Raeder, J., Frihagen, F., Ranhoff, A.H., et al., 2016. Increased CSF levels of aromatic amino acids in hip fracture patients with delirium suggests higher monoaminergic activity. BMC Geriatr. 16, 149.

Watne, L.O., Torbergsen, A.C., Conroy, S., Engedal, K., Frihagen, F., Hjorthaug, G.A., et al., 2014b. The effect of a pre- and postoperative orthogeriatric service on cognitive function in patients with hip fracture: randomized controlled trial (Oslo Orthogeriatric Trial). BMC Med. 12, 63.

World Health Organization, 1993. The ICD-10 Classification of Mental and Behavioural Disorders Diagnostic criteria for research. http://www.who.int/classifications/icd/en/GRNBOOK.pdf.

Wyller, T.B., Watne, L.O., Torbergsen, A., Engedal, K., Frihagen, F., Juliebo, V., et al., 2012. The effect of a pre- and post-operative orthogeriatric service on cognitive function in patients with hip fracture. The protocol of the Oslo Orthogeriatrics Trial. BMC Geriatr. 12, 36.

Zarow, C., Lyness, S.A., Mortimer, J.A., Chui, H.C., 2003. Neuronal loss is greater in the locus coeruleus than nucleus basalis and substantia nigra in Alzheimer and Parkinson diseases. Arch. Neurol. 60(3), 337-341.

FIGURE LEGENDS

Figure 1

Figure 1 heading: CSF catecholamine levels were analyzed in three cohorts.

Figure 1 legend: A) The hip fracture cohort included patients with and without demenita and delirium. Information about delirium at any time was missing for one patient and delirium status when CSF was obtained was missing for four patients. These patients were excluded from analyses requiring this information. B) The medical delirium cohort included patients all with delirium due to another medical condition while C) the final cohort included cognitively normal elderly patients.

Figure 2

Figure 2 heading: CSF catecholamine levels in patients with and without delirium in all cohorts. Figure 2 legend: A) CSF noradrenaline levels and B) CSF adrenaline levels were higher among the hip fracture patients (all, n=118) while C) CSF dopamine levels were highest among the cognitively normal elderly (n=122). Furthermore hip fracture patients with delirium (n=65) had compared those with no delirium (n=52) B) lower CSF adrenaline levels and C) lower CSF dopamine levels. Medical delirium patients (n=26) also had lower CSF dopamine relative to the cognitively normal elderly. CSF: Cerebrospinal fluid. Larger and smaller lines represent median and interquartile range respectively. Two-tailed p-values are for comparison of two groups are and obtained by Mann-Whitney U test. Differences in the three cohorts (upper line) except cohortwere analyzed by differences for which Kruskal-Wallis test was applied.

Figure 3

Figure 3 heading: CSF catecholamine levels in hip fracture patients with and without pre-fracture dementia.

For Review Only

Figure 3 legend: Hip fracture patients with pre-fracture dementia (n=60) did compared to those with no pre-fracture dementia (n=58) A) not have statistically significant lower CSF noradrenaline levels (p=0.17) but had significantly lower CSF levels of B) adrenaline and C) dopamine. CSF: Cerebrospinal fluid. Larger and smaller lines represent median and interquartile range respectively. Two-tailed p-values are obtained by Mann-Whitney U test.

Figure 4

Figure 4 heading: CSF catecholamine levels in delirium sub-grouped according to pre-fracture dementia status.

Figure 4 legend A-C) Among patients without pre-fracture dementia A) CSF noradrenaline was although not significant higher in incident delirium (n= 7) compared to those with no delirium (n=43; p=0.08). D-F) There were no significant differences among pre-fracture dementia patients. CSF: Cerebrospinal fluid. Larger and smaller lines represent median and interquartile range respectively. Two-tailed p-values are obtained by Mann-Whitney U test.

For Review Only

For Review Only

Table 1 Background characteristics of the three cohorts according to delirium status.

	1.Cognitively normal	2. Medical delirium	Hip fracture			p			
			3. All	4. No delirium	5. Delirium	Group 1-3	Group 2 vs 5	Group 4 vs 5	Group 1 vs 2
N	*122*	*26*	*118*	*52*	*65*				
Age	71.0 (68-76)	67.5 (61-77)	85 (80-89)	84(72-88)	85(81-90)	<0.001	<0.001	0.04	0.04
Gender									
Male	62 (50.8)	16 (61.5)	33 (28.0)	12(23.1)	21 (32.3)				
Female	60 (49.2)	10 (38.5)	85 (72.0)	40(72.9)	44 (67.7)				
Delirium									
No	122 (100)	0 (0)	52 (44.4)	52(100)	0(0)				
Yes	0 (0)	100 (100)	65 (55.6)	0 (0)	100(100)				
Dementia									
No	122 (100)	17 (65.4)	58 (49.6)	83%	77%				
Yes	0 (0)	9 (34.6)	60 (50.4)	17%	23%				
CSF catechols									
Noradrenaline	13.4 (9.8-20.1)	17.8 (8.5-24.5)	38.2 (24.6-53.3)	39.4(28.4-51.7)	35.4(21.8-53.5)	<0.001	<0.001	0.21	0.30
Adrenaline	5.9 (3.9-8.3)	6.0 (3.6-7.4)	8.4 (5.3-12.7)	10.4(6.7-13.6)	7.9(4.5-12.0)	<0.001	0.049	0.03	0.96
Dopamine	2.6 (1.8-3.2)	1.2 (0.8-2.0)	1.5 (0.8-2.2)	2.0(1.2-2.8)	1.3(0.6-1.8)	<0.001	0.49	0.002	<0.001

Data are presented as number with percentages in brackets for gender, delirium and dementia. Age (years) and CSF catecholamine levels (nM) are presented as median and interquartile ranges as shown by the 25 and 75 percentiles. 2-tailed p-values are obtained by Kruskal-Wallis-test for comparisons across the cohorts while Mann-Whitney U test is applied for delirium group comparisons.

Table 2: Background characteristics; hip fracture patients with and without dementia

NO PRE-FRACTURE DEMENTIA

	**All**	**No Delirium**	**Delirium**		
			**All**	**Incident**	**Prevalent**
N	*58*	*43*	*15*	*7*	*8*
Age	84 (78-88)	84 (72-88)	85 (81-88)	86 (81-92)	85(80-88)
Sex					
Male	17	11	6	3	3
Female	41	32	9	4	5
Time to surgery[1]	23 (16-34)	23 (14-31)	27 (21-36)	22 (13-32)	30(22-37)
APACHE score[2]	8 (7-10)	8 (7-10)	9 (6-12)	8 (6-15)	10(7-12)
CSF					
Noradrenaline	39.7 (30.4-53.7)	38.5 (27.8-49.9)	41.4 (34.6-66.4)	42.5 (40.9-72.4)	35.5(25.2-51.5)
Adrenaline	10.6 (6.9-13.4)	10.6 (7.1-13.6)	10.6 (6.2-12.3)	12.0 (5.0-19.7)	8.6(6.4-10.9)
Dopamine	2.0 (1.3-2.8)	2.0 (1.3-2.8)	1.8 (1.4-3.2)	2.3 (1.5-3.3)	1.6(0.6-2.6)

PRE-FRACTURE DEMENTIA

	**All**	**No Delirium**	**Delirium**		
			**All**	**Incident**	**Prevalent**
N	*60*	*9*	*50*	*13*	*33*
Age	86 (81-90)	86 (71-92)	85 (81-90)	87 (84-91)	85(80-88)
Sex					
Male	16	1	15	5	8

For Review Only

Female	44		8		35		8		25	
Time to surgery[1]	26	(13-43)	28	(16-34)	26	(13-44)	18	(10-28)	38	(22-46)
APACHE score[2]	9	(8-10)	7	(6-9)	9	(8-10)	8	(7-9)	9	(8-11)
CSF										
Noradrenaline	35.2	(19.5-52.9)	47.1	(32.9-63.9)	30.3	(19.1-51.8)	30.8	(19.2-53.4)	30.6	(18.7-51.6)
Adrenaline	7.4	(4.2-11.6)	9.8	(4.8-15.4)	7.4	(4.1-10.5)	7.9	(4.7-12.9)	6.3	(4.0-11.0)
Dopamine	1.1	(0.6-1.8)	2.0	(0.7-2.5)	1.0	(0.6-1.7)	0.6	(0.5-2.1)	1.1	(0.7-1.6)

Data are presented as median and interquartiles. CSF noradrenaline, adrenaline and dopamine are given in nM while age and time to surgery in years and hours respectively. Information about delirium status was missing for one patient and delirium status when CSF was obtained was missing for four patients. [1]Time to surgery; hours from hospital admission to surgery [2]APACHE score without blood-gas

For Review Only

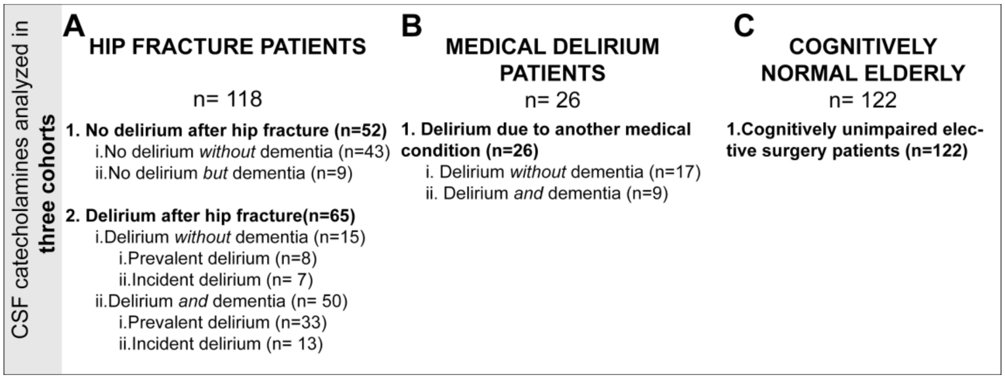

237x88mm (300 x 300 DPI)

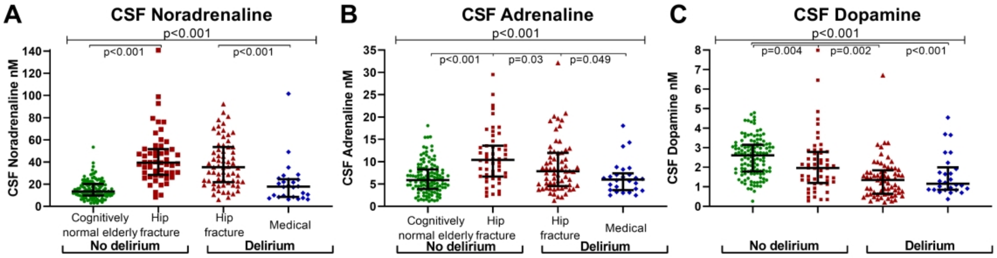

Figure 2 heading: CSF catecholamine levels in patients with and without delirium in all cohorts. Figure 2 legend: A) CSF noradrenaline levels and B) CSF adrenaline levels were higher among the hip fracture patients (all, n=118) while C) CSF dopamine levels were highest among the cognitively normal elderly (n=122). Furthermore hip fracture patients with delirium (n=65) had compared those with no delirium (n=52) B) lower CSF adrenaline levels and C) lower CSF dopamine levels. Medical delirium patients (n=26) also had lower CSF dopamine relative to the cognitively normal elderly. CSF: Cerebrospinal fluid. Larger and smaller lines represent median and interquartile range respectively. Two-tailed p-values are and obtained by Mann-Whitney U test except cohort differences for which Kruskal-Wallis test was applied.

210x60mm (600 x 600 DPI)

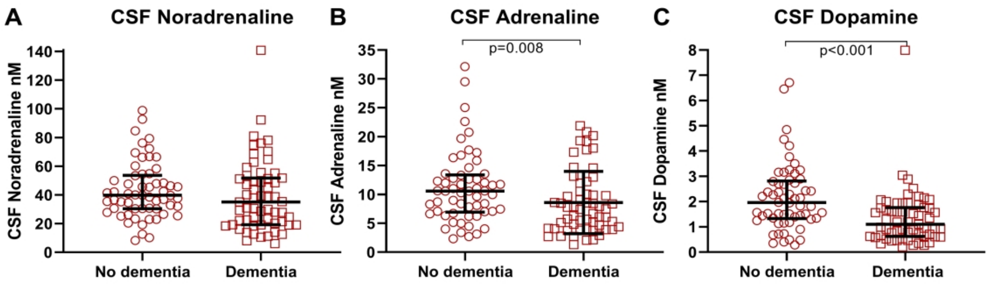

Figure 3 heading: CSF catecholamine levels in hip fracture patients with and without pre-fracture dementia. Figure 3 legend: Hip fracture patients with pre-fracture dementia (n=60) did compared to those with no pre-fracture dementia (n=58) A) not have statistically significant lower CSF noradrenaline levels (p=0.17) but had significantly lower CSF levels of B) adrenaline and C) dopamine. CSF: Cerebrospinal fluid. Larger and smaller lines represent median and interquartile range respectively. Two-tailed p-values are obtained by Mann-Whitney U test.

210x58mm (600 x 600 DPI)

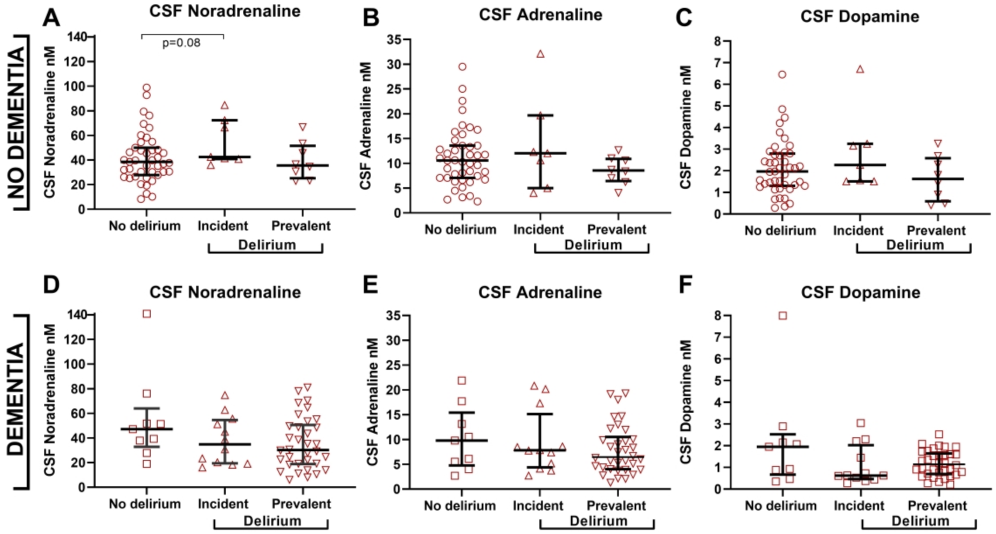

Figure 4 heading: CSF catecholamine levels in delirium sub-grouped according to pre-fracture dementia status.

Figure 4 legend A-C) Among patients without pre-fracture dementia A) CSF noradrenaline was although not significant higher in incident delirium (n= 7) compared to those with no delirium (n=43; p=0.08). D-F) There were no significant differences among pre-fracture dementia patients. CSF: Cerebrospinal fluid. Larger and smaller lines represent median and interquartile range respectively. Two-tailed p-values are obtained by Mann-Whitney U test.

207x116mm (600 x 600 DPI)

Supplementary data "Cerebrospinal fluid catecholamines in delirium and dementia"

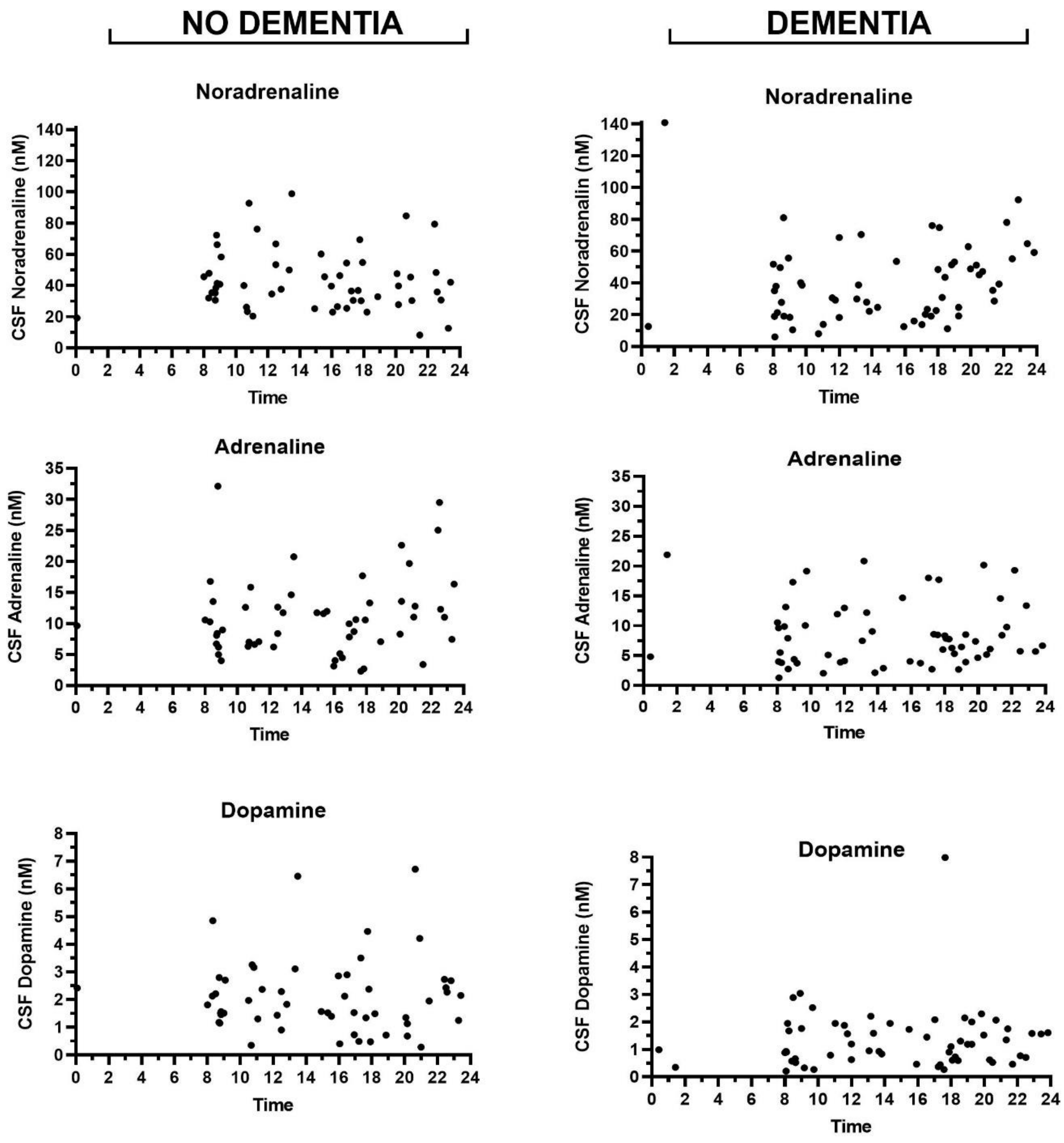

Supplementary Fig.S1: CSF catecholamines and time of day (time) of hip fracture surgery (time of lumbar puncture). There were no apparent diurnal fluctuations in the CSF catecholamine levels either in patients with pre-fracture dementia or without pre-fracture dementia.

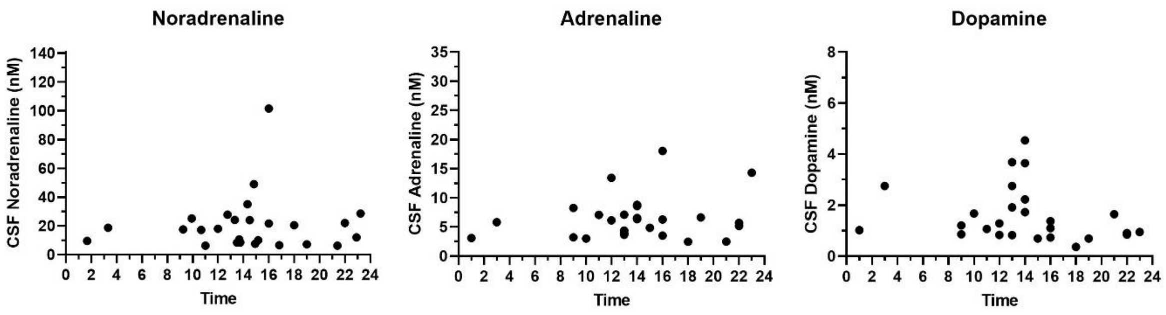

Supplementary Fig.S2: CSF catecholamines and time of day for lumbar puncture (time) among medical delirium patients. There were no apparent diurnal fluctuations in the CSF catecholamine levels for the medical delirium patients.

For Review Only
