# Supplementary material for: Cerebrospinal fluid catecholamines in delirium and dementia
Source: Brain Commun. 2021 May 29;3(3):fcab121. doi: 10.1093/braincomms/fcab121 (PMC8374970; doi:10.1093/braincomms/fcab121)
Supplement: fcab121_Supplementary_Data [file fcab121_Supplementary_Data.zip › Supplementary_material.pdf]

# Supplementary data: Supplementary Figure 1 and Supplementary Figure 2

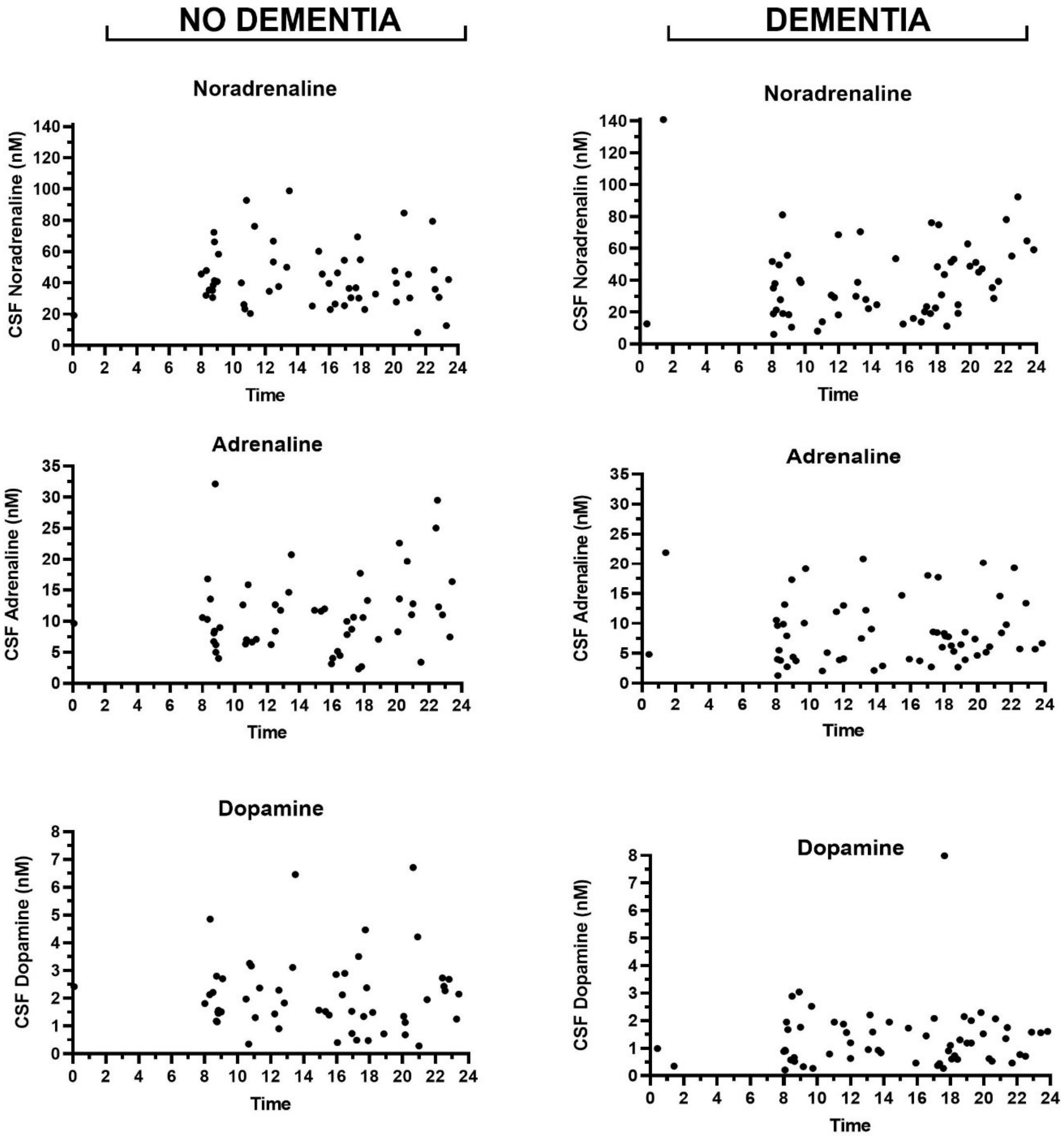


**Supplementary Figure 1: CSF catecholamine levels and time of day (time) of hip fracture surgery** (time of lumbar puncture). Analyses of CSF samples collected within the same 6 hour intervals (0:01 – 06:00, 06:01 – 12:00, 12:01 – 18:00, 18:01 – 24:00) showed no significant differences in the CSF catecholamine levels (all p = 0.51, 0.72 and 0.81, Kruskal-Wallis test, n= 115).

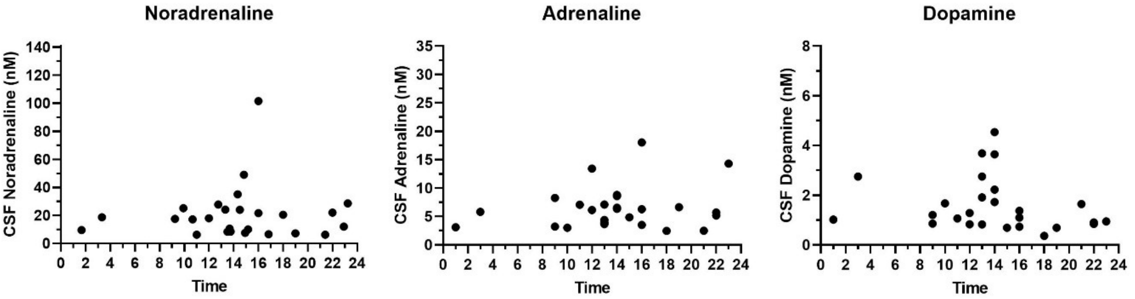


**Supplementary Figure 2: CSF catecholamines and time of day for lumbar puncture (time) among medical delirium patients.** Analyses of CSF samples collected within the same 6 hour intervals (0:01 – 06:00, 06:01 – 12:00, 12:01 – 18:00, 18:01 – 24:00) showed no significant differences in the CSF catecholamine levels (p = 0.78, 0.75 and 0.51, Kruskal-Wallis test, n= 26).
