# Supplementary material for: Cerebrospinal fluid catecholamines in delirium and dementia
Source: Brain Commun. 2021 May 29;3(3):fcab121. doi: 10.1093/braincomms/fcab121 (PMC8374970; doi:10.1093/braincomms/fcab121)
Supplement: fcab121_Supplementary_Data [file Original Submission-Decision Letter.pdf › Original Submission-Decision Letter.pdf]

Original Submission-Decision Letter

01-Oct-2020

Dear Dr. Henjum,

Thank you for your recent submission BRAINCOM-2020-227 entitled "Cerebrospinal fluid catecholamines in delirium and dementia" to Brain Communications.

Your manuscript has been reviewed, and while the reviewers and editorial team appreciate many aspects of the paper, they raise some concerns that you will need to address in order for the paper to be considered for publication. While I appreciate that it my not be possible to add pre-surgical CSF analyses from these patients, please be sure to include this as a clear limitation of the study and address all of the other comments including the requested power analysis.

If you can address these concerns, I invite you to submit a revised manuscript and a response to each of the reviewers' comments. The reviewers’ comments are included at the bottom of this message.

In addition to the reviewers' comments, please note our editorial comments added below to comply with our requirements for presentation of scientific data at Brain Communications.

Please format your revised manuscript according to our Instructions to Authors https://academic.oup.com/braincomms/pages/General_Instructions – a summary of key points is attached to help with this.

To revise your manuscript, click the link below or log into https://mc.manuscriptcentral.com/braincom and enter your Author Centre, where you will find your manuscript title listed under "Manuscripts with Decisions." Under "Actions," click on "Create a Revision." Your manuscript number has been appended to denote a revision.

*** PLEASE NOTE: This is a two-step process. After clicking on the link, you will be directed to a webpage to confirm. ***

https://mc.manuscriptcentral.com/braincom?URL_MASK=22503d9c9b2841b0814083e0a3f0a49d

Please upload a revised manuscript with changes tracked with track changes or in coloured text.

When submitting your revised manuscript, you will be asked to respond to the reviewers' comments. Please be as specific as possible in your response to all of the comments.

The editorial team at Brain Communications aims to increase transparency in scientific publishing and to demonstrate the value that peer review brings to the scientific process. Please therefore consider whether you are willing to have reviewers’ comments on your paper and your response published as supplementary data with the paper if it is accepted. This is entirely optional and will only be done if both the authors and reviewers agree. While submitting your revision there will be an opportunity to review or update the answer you gave previously, if necessary.

IMPORTANT: Your original files are available to you when you upload your revised manuscript. Please delete any redundant files before completing the submission.

Because we are trying to facilitate timely publication of manuscripts submitted to Brain Communications, we would appreciate your revised manuscript as soon as possible. If it is not possible for you to resubmit within 3 months, the option to submit a revision will expire, and we would then consider your revised paper as a new submission. However, if you need a little more time, please get in touch and we can extend this deadline for you.

Once again, thank you for submitting your manuscript to Brain Communications and I look forward to receiving your revision.

Yours sincerely,

Prof. Tara Spires-Jones
Editor, Brain Communications

Reviewer(s)' Comments to Author:

Reviewer: 1

Comments to the Author
The manuscript is generally well written, with clearly stated hypotheses, and is valuable for contributing data on the relationship between post-operative delirium, dementia, and CSF catecholamine concentrations. Lumbar CSF was collected from three groups (cognitively normal elderly patients (n=122), hip fracture patients with or without delirium and dementia (n=118), and other delirium, n=26). Delirium was assessed by standard method and exclusion criteria for confounding factors of medication and relevant co-morbidities were employed. Results showed that:

1) CSF Noradrenaline and adrenaline were raised in hip fracture versus cognitively normal and other delirium.
2) CSF dopamine was decreased in all hip fracture and all delirium versus cognitively normal.
3) CSF adrenaline and dopamine were decreased in hip fracture delirium versus hip fracture no delirium.
4) CSF adrenaline and dopamine were decreased in dementia versus no dementia subsets of the hip fracture group.
5) Sub-setting dementia and delirium incidence did not reveal significant differences.

Data was non-parametric and analysed by appropriate methods. The authors conclude that the results do not support the use of antipsychotic drugs for delirium patients.

Analyte measurement was conducted by liquid chromatography utilising single point calibration, which enables concentration calculation, and an internal DHBA standard, which allows for concentration normalisation across samples. My understanding is that single point calibration produces a point that is the response of the calibrant and a value for the slope of the regression line which is assumed to pass through x=0, y=0, but which should be demonstrated. Please expand the relevant methods section to demonstrate that the calibration method was validated, and describe how the raw data were treated to produce concentration values.

Dementia in the cohort is not well described. Please include the forms and representation of dementia present in the cohort.

The CSF collection period for hip fracture cohorts is described as “all hours” (P8, line 33:), and is not given for cognitively normal as far as I can see. For other delirium, collection is only described as ranging between 09:00 and 18:00 in 19 of the 26 patients. The methods section should include a more complete description of CSF collection period for all groups.

CSF noradrenaline was shown not to be influenced by diurnal rhythmicity, but what about adrenaline and dopamine? Given other studies have shown diurnal variation in all CSF catecholamines, demonstration that all molecules measured were not affected would be appropriate to justify excluding sample timing as a variable.

Age and sex disparities between groups reflexively cause me concern. However, the authors provide satisfactory evidence that these variables did not meaningfully impact results. The effect of sex in the medical delirium group is not mentioned (it would be good to do this alongside discussion of the other groups), though one can infer there was an effect as sex had to be accounted for in later analysis.

The authors conduct an interesting breakdown of dementia and delirium incidence. The overlap of dementia with delirium imbalanced group n between no delirium and delirium groups, and sub-setting by incidence decreased group n, making it difficult to draw meaningful conclusions. The authors acknowledge reduced statistical power as a limitation of this analysis, but it would be helpful to know whether it met commonly used power criteria (alongside the results) rather than inferring that it did not.

Recommend referencing the following publication in the context of discussing post-operative delirium and dementia: Cunningham et al. CSF Beta-amyloid 1-42 Concentration Predicts Delirium Following Elective Arthroplasty Surgery in an Observational Cohort Study, Ann Surg, 2019

P7, line 54: “RS:- 0.05-0.05 with all p-values> 0.5, n=122)”. This is not expressed the same way as results just prior and confused me a little. I assume 0.05-0.05 represents range of results from all three catecholamines, but as they seem to all have been the same I think it would be more intuitive to state this, or present each value respectively as done previously.

P3:18 “dementia(Watne et al., 2016,” space required.

P3:33 “Brain adrenergic neurons locates to a few brainstem areas activated by stress (Guyenet et al., 2013).” Grammar.

P6:31 “Further the HPLC conditions were sat to:”. Spelling.

P9:31 Patients with pre-fracture dementia had compared to those without pre-fracture dementia, lower…”. Grammar.

Reviewer: 2

Comments to the Author
This study evaluated CSF levels of catecholamines in delirium and dementia. Authors compared different cohorts, including cognitive normal, hip fracture patients with or without dementia or delirium, and patients with medical delirium. Dynamic changes in CSF neurotransmitters were described. The study is interesting, although limited and further details should be provided:
1) a power analyses should be provided. Although the initial trial numbers are compelling for such study, authors further sub-divided patients with different etiologies, shrinking the comparison pool. A statistical review should be added and addressed for the multiple comparisons here provided. Also, why including medical delirium in this study? Its etiology is very different from POD...
2) It seems CSF was collected once in the surgical cohort and compared to cognitive normal CSF. Not having a CSF baseline (pre-surgery) is a MAJOR limitation of the study and should be clearly addressed. Stress and other PRE-operative factors are likely to affect these biomarkers. A single timepoint evaluation is a limiting factor.
3) Was any data on sleep hygiene collected in these subjects? Poor sleep as often found in the hospital setting may be a strong confounder here.
4) were any patients diagnosed with delirium superimposed on dementia?

Editorial Office comments:
-Please supply a graphical abstract. A graphical abstract is a visual representation of the central finding or methodology of the paper. It will be displayed in the table of contents. Authors should strive to make the graphical abstract informative, interesting, visually appealing, and straightforward (no caption needed, no abbreviations except gene names). Please upload as a separate high resolution image file (tiff preferred). The technical requirements for the graphical abstracts are:
- size of the submitted image: 1200 pixels square at 300dpi
- font of the text: Arial 12-16 point (smaller fonts will not be legible online)
