# Supplementary material for: Cerebrospinal fluid catecholamines in delirium and dementia
Source: Brain Commun. 2021 May 29;3(3):fcab121. doi: 10.1093/braincomms/fcab121 (PMC8374970; doi:10.1093/braincomms/fcab121)
Supplement: fcab121_Supplementary_Data [file Original Submission-Response to Reviewers.pdf › Original Submission-Response to Reviewers.pdf]

Regarding the manuscript BRAINCOM-2020-227 entitled "Cerebrospinal fluid catecholamines in delirium and dementia". We would like to thank the reviewers for their careful examination of our manuscript and valuable comments. The comments of the reviewers are addressed below.

REVIEWER 1

We thank for your critical examination of the manuscript. Please see our response to your comments with manuscript revisions shown in *italics*.

Reviewer 1 Commment #1:

Analyte measurement was conducted by liquid chromatography utilising single point calibration, which enables concentration calculation, and an internal DHBA standard, which allows for concentration normalisation across samples**. My understanding is that single point calibration produces a point that is the response of the calibrant and a value for the slope of the regression line which is assumed to pass through x=0, y=0, but which should be demonstrated.** Please expand the relevant methods section to demonstrate that the **calibration method was validated**, and describe how the **raw data were treated to produce concentration values**.

Response reviewer 1 comment #1

We thank the reviewer for this relevant comment, and acknowledge the importance and relevance for a methodical explanation and of extending and clarified the method part.

Analyzes of plasma catecholamines parameters (noradrenalin, adrenaline and dopamine) is a well-established HPLC-ECD method. We have used commercial assay reagents from Chromsystem GmbH (Catecholamines in Plasma – HPLC) to perform this analysis for several years, and also for this specific project. Here, we customized the method for measuring catecholamines in CSF. Several studies have shown that plasma and CSF matrix is comparable in the case of component with validation of both, the response ratio and peak separation.

In the current study we applied calibration standard contains 3 catecholamine metabolites (Noradrenalin, Adrenalin and Dopamine), and an internal standard (DHBA) correcting for deviations in the injector and the detector. In ChemStation (software version B.04.03 SP2 [108]) the response factor (sample intensity/DHBA) is plotted as a single-point calibration, since the regression curve is found to be linear. Single-point calibration use one level (one calibration standard), which is the highest level of quantification. All the unknown concentrations are found by the factor (slope) ChemStation calculate. The theory for ECD assumes that the intensity (concentration) of the sample is linear over the working range. The CSF concentration of an unknown sample is calculated by the linear calibration curve (y = ax+b). The ECD settings used are working potential 0.60V and the range 50nA. The CSF was based on the plasma method from Chromsystem GmbH which was validated with linearity through the origo (x=0, y=0). This would also apply here as comparison of CSF to plasma matrix has demonstrated that these are compatible biologic matrixes in the HPLC-ECD method. Furthermore quality controls (a CSF control pool) was injected for every analytical series of samples, and during the analytical series every tenth injections. Hereby the linearity and the intercept were confirmed. Moreover, the CSF pool works as a control of any displacement of the retention time. We therefore consider single point calibration with a regression line passing through origo appropriate for the current analyzes.

We have revised the method section of the manuscript paragraph “CSF catecholamine analyses”:

CSF samples were directly injected in the HPLC-ECD system (no CSF pre-preparation with solid-phase extraction- or sample clean up columns) and run after the calibration standards. Peak for each component were integrated and identified from calibration retention time and the peak heights related to a concentration from the same standard. *Single point calibration with a regression line through origo is shown appropriate for plasma catecholamines by the reagent supplier Chromsystem GmbH. CSF and plasma have comparable matrixes in HPLC-ECD analyzes (Hooshfar et al., 2016) and single point calibration was applied for the current CSF analyses.*

Quality controls (a CSF control pool) was injected for every tenth injection. The inter-assay coefficients of variation (CV; based on measurements of pooled CSF samples repeated in each assayed series), were 4.7%, 6.2% and 8.3% for noradrenaline, adrenaline and dopamine, respectively.

Reviewer 1 #Comment 2

Dementia in the cohort is not well described. Please include the forms and representation of dementia present in the cohort.

Response Comment 2

Since hip fracture is an acute event, objective cognitive testing prior to the fracture was not possible. Diagnosis of dementia was therefore set by experienced geriatricians that had access to all clinical information with high agreement between them (Wyller *et al.*, 2012; Watne *et al.*, 2014). IQCODE is a validated and much used tool to assess for dementia status in acutely admitted patients, and we believe our approach with a consensus based dementia diagnosis is superior to the use of IQCODE alone. Nevertheless, the retrospective classification of dementia is a weakness of our study. We have no information regarding dementia etiology. In the medical delirium population IQCODE was not collected, and the dementia diagnoses had to be set from the hospital records.

We have therefore revised the manuscript methods section “Study participants, Hip fracture cohort”: *Based on all available clinical information* pre-fracture dementia status was determined by consensus in an expert panel. The International Classification of Diseases (ICD)-10 criteria (World Health Organization, 1993) *was applied for dementia diagnosis without further information on dementia etiology*.

We have also included this a limitation of the study in the discussion:
The retrospective classification of dementia without further information on dementia etiology is a limitation of the study. People with Dementia with Lewy bodies (DLB) may have higher CSF noradrenaline levels than other dementias and a higher risk of delirium (Janssens et al., 2018). However few patients would be expected to have DLB as Alzheimer’s disease dementia with mixed pathology is the most common cause of dementia in aged populations (Barker et al., 2002; Jellinger and Attems, 2010).

Reviewer 1 # Comment 3

The CSF collection period for hip fracture cohorts is described as “all hours” (P8, line 33:), and is not given for cognitively normal as far as I can see. For other delirium, collection is only described as ranging between 09:00 and 18:00 in 19 of the 26 patients. The methods section should include a more complete description of CSF collection period for all groups.

Response Reviewer 1 # Comment 3

For the hip fracture patients CSF was collected in connection with the hip fracture surgery. As acute hip fracture surgeries are performed at all hours CSF was also collected at all hours. In contrast elective surgery were performed at daytime and all CSF samples of the cognitively unimpaired obtained at daytime. For the medical delirium patients' diagnostic lumbar puncture were performed at all hours. We agree that informing that most medical delirium patients underwent their diagnostic LP between 09-18 is sparse and that the CSF from the elective surgery patients is obtained during daytime should be clarified.

We have therefore revised the manuscript paragraph "CSF sampling and storage":

"*Hip fracture surgeries were performed at all hours (see Supplementary Fig .SF1). Elective surgeries were performed at daytime only and CSF from such patients were therefore all sampled at daytime. Diagnostic LP in the medical delirium patients was also performed at all hours but for most at daytime (01:30-03: 30: 2 patients, 09-12; 5 patients; 13:30-16: 13 patients 18-24; 6 patients* see *Supplementary Fig. SF2).*

Reviewer 1 Comment #4

CSF noradrenaline was shown not to be influenced by diurnal rhythmicity, but what about adrenaline and dopamine?

Given other studies have shown diurnal variation in all CSF catecholamines, demonstration that all molecules measured were not affected would be appropriate to justify excluding sample timing as a variable.

Response reviewer 1 Comment #4

We find the reviewers request of justification to exclude sample timing as a variable for all three catacholamines and not only noradrenaline well founded. Supplementary Figure S1 is revised to include all three catecholamines and time of day of CSF sampling in the hip fracture patients. We have also added Supplementary Figure S2 with time of day of lumbar puncture and CSF catecholamine levels for the medical delirium patients. We hope the reviewer and potential readers are ensured that excluding sample timing as a variable is appropriate

The manuscript results section "Catecholamine interrelations and relations to age, gender and diurnal rhythm" is revised with reference to the supplementary figures:

Acute hip fracture surgery and diagnostic LPs for the medical delirium patients were performed at all hours. The CSF catecholamines did not display diurnal rhythmicity neither in the hip fracture nor medical delirium patients (Supplementary Fig. S1 and S2). Age, and time of surgery or diagnostic LP were therefore not included in further analyses while gender were accounted for in relevant analyses.

Reviewer 1 # Comment 5

Age and sex disparities between groups reflexively cause me concern. However, the authors provide satisfactory evidence that these variables did not meaningfully impact results. The effect of sex in the medical delirium group is not mentioned (it would be good to do this alongside discussion of the other groups), though one can infer there was an effect as sex had to be accounted for in later analysis.

Response reviewer 1 Comment #5

The gender distribution was somewhat different in the three cohorts (table 1). There were no significantly different gender distribution in the cognitively normal elderly (p=0.86) or in the medical delirium group (p=0.24). There were more females than males in the hip fracture cohort (p<0.001), but gender did not impact catecholamine levels. Since this was the main cohort in our study, we suggest to keep this information in the manuscript.

We found it appropriate to also examine the effect of gender on catecholamine levels in the cognitively normal elderly as this cohort had patients without dementia and delirium (that potentially could impact catecholamine levels and therefore obscure an effect of gender), and the gender distribution was even. There was no effect of gender on noradrenaline or adrenaline, but the cognitively normal females had higher CSF dopamine levels (p=0.03). Therefore, for analyses of CSF dopamine gender-separated analyses were performed.

#Reviewer 1 Comment 6

The authors conduct an interesting breakdown of dementia and delirium incidence. The overlap of dementia with delirium imbalanced group n between no delirium and delirium groups, and sub-setting by incidence decreased group n, making it difficult to draw meaningful conclusions. The authors acknowledge reduced statistical power as a limitation of this analysis, but it would be helpful to know whether it met commonly used power criteria (alongside the results) rather than inferring that it did not.

Response Reviewer 1 Comment 6

As the reviewer point out the overlap between dementia and delirium challenge the data interpretation. Although the studies of delirium pathophysiology are increasing, studies on CSF biomarkers are still sparse, reflecting the challenges of obtaining CSF from such patients. Our CSF sample set is among the largest but we acknowledge limited statistical power in stratified analyses. Dementia is the strongest risk factor of delirium, and might impact the same biomarkers as delirium. While waiting for larger cohorts to be collected, we therefore think it is worth reporting results from analysis stratified on dementia status in our cohort, even if the number of individuals in some of the sub-analyses were limited.

CSF catecholamines have not been examined in the context of delirium before and the current study should therefore be regarded as explorative with need for replication. Performing power calculations before the study to detect if the number of participants with CSF available was sufficient would be challenged by an unknown expected effect size. *Performing post hoc* power tests is not recommended (Lydersen, 2015). As number of individuals in some of the sub-analyses were limited, we acknowledged this by listing this as a limitation as this increase the chance of both type I and type II errors (Button *et al.*, 2013).

Reviewer 1 Comment 7

Recommend referencing the following publication in the context of discussing post-operative delirium and dementia: Cunningham et al. CSF Beta-amyloid 1-42 Concentration Predicts Delirium Following Elective Arthroplasty Surgery in an Observational Cohort Study, Ann Surg, 2019

Response Reviewer 1 Comment 7

Thank you for your input. We have revised the manuscript and supplemented it with this relevant information in the discussion; "*Already in the preclinical phase do Alzheimer's disease seem to pose an increased risk of delirium (Idland et al., 2017; Cunningham et al., 2019)*."

Reviewer 1 Comment 8

P7, line 54: "RS:- 0.05-0.05 with all p-values> 0.5, n=122)". This is not expressed the same way as results just prior and confused me a little. I assume 0.05-0.05 represents range of results from all three catecholamines, but as they seem toall have been the same I think it would be more intuitive to state this, or present each value respectively as done previously.

P3:18 "dementia(Watne et al., 2016," space required.

P3:33 "Brain adrenergic neurons locates to a few brainstem areas activated by stress (Guyenet et al., 2013)." Grammar.

P6:31 "Further the HPLC conditions were sat to:". Spelling.

P9:31 Patients with pre-fracture dementia had compared to those without pre-fracture dementia, lower…". Grammar.

Response Reviewer 1 comment 8.

Thank you for your input it is certainly in our interest to present the data clearly. We apologize the grammar errors and have revised these in the manuscript. Regarding " Rs:- 0.05-0.05 with all p-values> 0.5, n=122) the reviewers assumption is correct. This is now revised and the three Rs reported for clarity and consistency: *"Rs: 0.05, -0.04 and -0.07 respectively with all p-values>0.5, n=122)."* The Rs for dopamine -0.068 is slighly below -0.05 and we sincerely apologize for this error.

REVIEWER: 2

Comments to the Author

This study evaluated CSF levels of catecholamines in delirium and dementia. Authors compared different cohorts, including cognitive normal, hip fracture patients with or without dementia or delirium, and patients with medical delirium. Dynamic changes in CSF neurotransmitters were described.

We are happy to hear that reviewer find this study of CSF catecholamines in delirium interesting and thank the reviewer for the assessment of the manuscript and valuable input for improvement. Please find our response to your comments below. Manuscript revisions are given *in italics*.

The study is interesting, although limited and further details should be provided:

1) a power analyses should be provided. Although the initial trial numbers are compelling for such study, authors further sub-divided patients with different etiologies, shrinking the comparison pool. A statistical review should be added and addressed for the multiple comparisons here provided. Also, why including medical delirium in this study? Its etiology is very different from POD...

Response reviewer 2 comment #1

1a) Regarding power analyses we also responded to reviewer 1 comment 7; CSF studies of delirium is rare and this is to our knowledge one of the first study reporting CSF catecholamine levels in delirium. The study is thereby explorative and the effect size for calculating sample size was not known. Performing post-hoc power calculations is however not recommended (Lydersen, 2015). Still given the intra-group variation observed we think it is appropriate to report limited study power in the smaller groups as a limitation of the study as this both increase the chance of performing type I but also type II errors (Button *et al.*, 2013).

1b) About multiple comparisons. Three cohorts were included in this study of CSF catecholamines; a hip fracture cohort (case/control), a medical delirium cohort (cases) and a cohort of cognitively normal elderly (controls). The trauma of the hip fracture was expected to induce several responses. Therefore the cognitively normal patients were included as an appropriate control group for the medical delirium patients while hip fracture patients without delirium was the intended reference group for the hip fracture patients with delirium.

We understand that the multiple comparisons might reduce readability of our paper. On the other hand we feel it is important to show all the different cohorts, since the hip fracture trauma itself seems to impact catecholamine levels.

1c) About inclusion of the medical delirium patients. The aim of this study was to examine CSF catecholamines in delirium. Hip fracture patients comprised the main study population. The medical delirium patients were included as a group with non-surgical precipitated delirium. As pointed out by the reviewer these patients form a group with a different precipitating factor of delirium than the hip fracture patients. A goal of a study like ours would be to understand changes in biomarker levels associated with delirium in itself, and what changes can be explained by precipitating factors of delirium (hip fracture, sepsis, pneumonia etc). Thus the medical delirium group served as a second delirium group included to see if the CSF catecholamine levels observed in the hip fracture patients with delirium were specific to this patient cohort or similar patterns would emerge in the medical delirium group.

We have therefore revised the manuscript (#comment 1a-c):

Introduction

We asked if the CSF catecholamine levels are altered in delirium. *To answer this study question CSF* catecholamine levels were analyzed in *1)* hip fracture patients with and without delirium and dementia, *2)* in a second delirium group with delirium precipitated by another medical condition and in *3)* cognitively normal elderly patients as a reference.

Discussion study limitation included:

This may increase the likelihood of false negative but also false positive results *(Button et al., 2013)* (reference added). *As an explorative study the results should be confirmed in larger follow up studies (Althouse, 2016).*

Figure legend 2: *Two-tailed p-values for comparison of two groups are obtained by Mann-Whitney U test. Differences in the three cohorts (upper line) were analyzed by Kruskal-Wallis test.*

2) It seems CSF was collected once in the surgical cohort and compared to cognitive normal CSF. Not having a CSF baseline (pre-surgery) is a MAJOR limitation of the study and should be clearly

addressed. Stress and other PREoperative factors are likely to affect these biomarkers. A single timepoint evaluation is a limiting factor.

Response reviewer 1 comment #2

Indeed we agree with the reviewer that stress and other PREoperative factors are likely to affect these biomarkers. Hip fracture patients without delirium therefore served as a reference group for the hip fracture patients with delirium. Hip fracture patients that developed delirium before and after surgery was included. This allowed for a cross-sectional approach to study CSF changes before and after delirium was clinically present. We agree with the reviewer that longitudinal sampling would be preferable. However, such a study will be very hard to perform since patients will have to volunteer for an additional lumbar puncture without clinical indication. Most CSF biomarker studies in delirium are done on patients undergoing surgery, since this offer the opportunity to collect CSF in conjunction with administration of the anesthetic agents. To collect CSF on delirious patients for research purposes only will be ethical challenging. We hope the reviewer agree and find the study acceptable with the cross-sectional approach as applied.

The manuscript have been revised to include this as a study limitation:

The cross-sectional design has been included as a study limitation: *The cross-sectional design is a limitation of the study but longitudinal CSF delirium studies is challenging to perform.*

3) Was any data on sleep hygiene collected in these subjects? Poor sleep as often found in the hospital setting may be a strong confounder here.

Response reviewer 2 comment #3

Thank you for this relevant comment. Unfortunately we did not have information of sleep hygiene in this study.

The manuscript have been revised to include this a limitation in the manuscript in the discussion:
Poor sleep could also influence the study but could not be adjusted for which is another study limitation.

4) were any patients diagnosed with delirium superimposed on dementia?

Response reviewer 2 comment #4

Yes there were patients diagnosed with delirium superimposed on dementia (see Table 1 and 2). We have also included this in the text; "The majority of the hip fracture patients with delirium had delirium superimposed on dementia (~75%), while most patients without pre-fracture dementia did not develop delirium (~75%, Table 2)."

REFERENCES

Althouse, A.D., 2016. Adjust for Multiple Comparisons? It's Not That Simple. Ann. Thorac. Surg. 101(5), 1644-1645.

Button, K.S., Ioannidis, J.P., Mokrysz, C., Nosek, B.A., Flint, J., Robinson, E.S., et al., 2013. Power failure: why small sample size undermines the reliability of neuroscience. Nat. Rev. Neurosci. 14(5), 365-376.

Cunningham, E.L., McGuinness, B., McAuley, D.F., Toombs, J., Mawhinney, T., O'Brien, S., et al., 2019. CSF Beta-amyloid 1-42 Concentration Predicts Delirium Following Elective Arthroplasty Surgery in an Observational Cohort Study. Ann. Surg. 269(6), 1200-1205.

Hooshfar, S., Basiri, B., Bartlett, M.G., 2016. Development of a surrogate matrix for cerebral spinal fluid for liquid chromatography/mass spectrometry based analytical methods. Rapid Commun. Mass Spectrom. 30(7), 854-858.

Idland, A.V., Wyller, T.B., Stoen, R., Eri, L.M., Frihagen, F., Raeder, J., et al., 2017. Preclinical Amyloid-beta and Axonal Degeneration Pathology in Delirium. J. Alzheimers Dis. 55(1), 371-379.

Janssens, J., Vermeiren, Y., Fransen, E., Aerts, T., Van Dam, D., Engelborghs, S., et al., 2018. Cerebrospinal fluid and serum MHPG improve Alzheimer's disease versus dementia with Lewy bodies differential diagnosis. Alzheimer's & dementia (Amsterdam, Netherlands) 10, 172-181.

Lydersen, S., 2015. Statistical review: frequently given comments. Ann. Rheum. Dis. 74(2), 323-325.

Watne, L.O., Torbergsen, A.C., Conroy, S., Engedal, K., Frihagen, F., Hjorthaug, G.A., et al., 2014. The effect of a pre- and postoperative orthogeriatric service on cognitive function in patients with hip fracture: randomized controlled trial (Oslo Orthogeriatric Trial). BMC Med. 12, 63.

World Health Organization, 1993. The ICD-10 Classification of Mental and Behavioural Disorders Diagnostic criteria for research. http://www.who.int/classifications/icd/en/GRNBOOK.pdf.

Wyller, T.B., Watne, L.O., Torbergsen, A., Engedal, K., Frihagen, F., Juliebo, V., et al., 2012. The effect of a pre- and post-operative orthogeriatric service on cognitive function in patients with hip fracture. The protocol of the Oslo Orthogeriatrics Trial. BMC Geriatr. 12, 36.
