# Supplementary material for: Cerebrospinal fluid catecholamines in delirium and dementia
Source: Brain Commun. 2021 May 29;3(3):fcab121. doi: 10.1093/braincomms/fcab121 (PMC8374970; doi:10.1093/braincomms/fcab121)
Supplement: fcab121_Supplementary_Data [file Revision 1-Decision Letter.pdf › Revision 1-Decision Letter.pdf]

Revision 1-Decision Letter

29-Dec-2020

Dear Dr. Henjum,

Your manuscript has been reviewed, and while the reviewers and editorial team appreciate many aspects of the paper, they have remaining concerns that mean the paper is not suitable for publication in Brain Communications. The reviews are below. If you can provide the requested power calculations, quantification, and details of clinical diagnoses, we will consider a revision of the manuscript.

Please format your revised manuscript according to our Instructions to Authors https://academic.oup.com/braincomms/pages/General_Instructions – a summary of key points is attached to help with this.

To revise your manuscript, click the link below or log into https://mc.manuscriptcentral.com/braincom and enter your Author Centre, where you will find your manuscript title listed under "Manuscripts with Decisions." Under "Actions," click on "Create a Revision." Your manuscript number has been appended to denote a revision.

*** PLEASE NOTE: This is a two-step process. After clicking on the link, you will be directed to a webpage to confirm. ***

https://mc.manuscriptcentral.com/braincom?URL_MASK=3c9a69d8c15c45e6b08098a49a372c3f

Please upload a revised manuscript with the changes you have made shown either with "track changes" or in coloured text.

When submitting your revised manuscript, you will be asked to respond to the reviewer(s) comments. Please be as specific as possible in your response to all of the comments.

The editorial team at Brain Communications aims to increase transparency in scientific publishing and to demonstrate the value that peer review brings to the scientific process. Please therefore consider whether you are willing to have reviewer(s)' comments on your paper and your response published as supplementary data with the paper if it is accepted. This is entirely optional and will only be done if both the authors and reviewers agree. While submitting your revision there will be an opportunity to review or update the answer you gave previously, if necessary.

IMPORTANT: Your original files are available to you when you upload your revised manuscript. Please delete any redundant files before completing the submission.

Because we are trying to facilitate timely publication of manuscripts submitted to Brain Communications, we would appreciate your revised manuscript as soon as possible. If it is not possible for you to resubmit within 3 months, the option to submit a revision will expire, and we would then consider your revised paper as a new submission. However, if you need a little more time, please get in touch and we can extend this deadline for you.

Once again, thank you for submitting your manuscript to Brain Communications and I look forward to receiving your revision.

Best wishes,

Prof. Tara Spires-Jones
Founding Editor, Brain Communications

Reviewer(s)' Comments to Author:

Reviewer: 1

Comments to the Author
Liquid chromatography utilising single point calibration – The authors refer the reader to the product manufacturer, without giving a product catalogue number to verify the product, or lot number as performance can be batch specific. Furthermore, the manufacturer does not provide the required information for its products without one having registered an account. The information should either be given directly in the methods section or a clear and open access reference should be provided. This concern has not been satisfactorily addressed.

The request for power calculation raised by both reviewers has not been addressed.

The reworked supplementary figures present the data more clearly. However, it is simply stated that there was no diurnal effect without any statistical analysis provided to qualify this. In Fig.S2 Dopamine certainly looks like it fluctuates with CSF collection time… numbers will be required to convince the reader of the author's claims.

The revised section on CSF collection times, alongside the supplementary figures, is acceptable. However, 'daytime' is an unnecessarily vague term, please just state the time interval for the reader.

Gramma needs checking in many of the revised sections.

All other comments have been addressed. However, I am disappointed that the presence/absence of relevant dementia forms have not been confirmed, it would be surprising if this was not in the report/notes/data generated by the expert panel.

Reviewer: 2

Comments to the Author
The study has been marginally improved, however concerns remain about the statistical rigor and study design, which were poorly rebutted by the authors.
In particular, the justification for not having power calculations as "the first study reporting CSF catecholamine levels in delirium" is unacceptable. Estimates can be calculated based on the changes herein provided. The exploratory nature of the study should not be presented as a valid justification for the "n" used here. Also, I don't think this is the first study ever measuring catecholamines in delirium (PMID: 32211614), thus data could likely be extrapolated and compared with other literature of CSF changes in delirium patients (not necessarily limited to catecholamines).
The next major limitation is the cross-sectional approach. Not having baseline measurement, especially for catecholamines that are extremely sensitive to multiple pre-existing/pre-operative stressors, cannot be justified by invoking ethical principles. Several studies are able to obtain lumbar CSF as part of research studies with necessary consent forms, IRB approval, and safety measures to these subjects. It sure is a lot of work, but is doable and done my several groups worldwide. Thus, I consider this is a major flaw in the study design that precludes in my opinion from a full evaluation of these results.
