# Supplementary material for: Cerebrospinal fluid catecholamines in delirium and dementia
Source: Brain Commun. 2021 May 29;3(3):fcab121. doi: 10.1093/braincomms/fcab121 (PMC8374970; doi:10.1093/braincomms/fcab121)
Supplement: fcab121_Supplementary_Data [file Revision 1-Response_to_reviewers.pdf › Revision 1-Response_to_reviewers.pdf]

We would once more like to thank the reviewers for their careful examination of the manuscript and constructive comments. Please see our response to the specific questions raised by the reviewers below. Changes are indicated by track changes in the enclosed manuscript file.

Reviewer: 1
Comments to the Author:

<u>Reviewer 1 Comment #1</u>
Liquid chromatography utilising single point calibration – The authors refer the reader to the product manufacturer,without giving a product catalogue number to verify the product, or lot number as performance can be batch specific. Furthermore, the manufacturer does not provide the required information for its products without one having registered an account. The information should either be given directly in the methods section or a clear and open access reference should be provided. This concern has not been satisfactorily addressed.

Response:
Analyses of plasma catecholamines (noradrenaline, adrenaline and dopamine) by high performance liquid chromotography with electrochemical detection (HPLC-ECD) is a well-established method. We have used commercial assay reagents from Chromsystems kit "The Catecholamines in Plasma – HPLC order no 5000" to perform this analysis for several years (Pytte *et al.*, 2006; Nygaard *et al.*, 2019; Undrum Bergland *et al.*, 2021). These reagents were also here used for determination of CSF catecholamine levels, all from the same lot no (0318). As the reviewer points out Chromsystems require a login for extended product information. However registering an account is free of charge and the information is thereby be available to interested readers. The catecholamines were detected by the ECD. The ECD supplier Antec Scientific inform to use single point calibration for plasma catecholamines in their product information which is available at their webpages. We apologize that this not was clear in the previous manuscript. We agree with the reviewer that the manuscript should include the Chromsystem order and lot number. The manuscript is also revised to clarify that single point calibration also relates to the ECD.

The manuscript now reads (page 6, Materials and Methods, subheading CSF catecholamine analyses):

Mobile phase, calibration standard and internal standard reagents came from the same company, Chromsystem GmbH (order no. 5000, all CSF samples were measured with reagents from lot no 0318).

(…)

For quantitative results all series started with a single point calibration sample (catecholamine calibration standard) as informed by the suppliers (Chromsystem GmbH and Antec, Scientific) for quantification of plasma catecholamines. This was here applied as CSF and plasma have comparable matrixes in HPLC-ECD analyses (Hooshfar et al., 2016).

Reviewer 1 Comment # 2
The request for power calculation raised by both reviewers has not been addressed.

Response:
CSF samples from hip fracture patients were collected as part of the Oslo Ortogeriatric Trial (OOT). The OOT was a randomized clinical trial with a primary aim to evaluate the effect of an orthogeriatic service on delirium associated long-term cognitive decline. Power calculations were performed prior to the study for this purpose. A secondary aim of the OOT was to explore delirium pathophysiology by analyzing CSF biomarkers as delirium was closely monitored in the OOT and CSF could be collected from hip fracture patients undergoing spinal anesthesia. All patients with available CSF were included in the current study.

The role of post-hoc power calculations (observed power) is debated. Some argue for a role of such analyses to retrospectively examine if the study had sufficient power to detect the effect. However non-significant studies are usually underpowered while significant studies adequately powered. This is exemplified by the observed power for two of the comparisons performed in the current study. Hip fracture patients without dementia but with incident delirium had higher but not significant CSF noradrenaline ($p=0.07$) compared to those without delirium. Calculating the observed power of his test showed a power of 0.49 (49%). In contrast the comparison of CSF dopamine levels in hip fracture patients with and without delirium ($p<0.05$) had an observed power of 0.83 (83%). The observed power of these Mann-Whitney tests were calculated by GPower and Laplace distribution applied (Faul *et al.*, 2007). Such calculations are however of several reasons strongly advised against (Hoenig and Heisey, 2001; Lydersen, 2015; Zhang *et al.*, 2019). We therefore do not find it appropriate to retrospectively perform power calculations.

To emphasize that the CSF samples was collected as part of a previous study, and that a priori power calculations for the present study not was carried out we have revised the methods and the discussion.

The manuscript now reads (page 3-4, Materials and Methods, subheading Study participants):

Hip fracture cohort
Hip fracture patients were recruited from the Oslo Orthogeriatric Trial (OOT) that included patients admitted to Oslo University Hospital from 2009 to 2012. The OOT was a randomized controlled trial evaluating the effect of an orthogeriatric service on delirium associated long-term cognitive decline. A secondary aim of the study was to investigate delirium pathophysiology. For this purpose CSF was collected at the onset of spinal anesthesia. All CSF samples available from OOT were included in the current study. (…) For further details see the previous the previous publications (Wyller et al., 2012; Watne et al., 2014b).

(Page 11 Discussion):
Still, although a large study in the context of delirium, the number of individuals in some subgroup analyses was limited. This may increase the likelihood of false negative but also false positive results (Button et al., 2013). All available samples collected in a previous study (the OOT) were included, a priori power calculations were not performed. As an explorative study the results should be confirmed larger follow up studies (Althouse, 2016).

Reviewer 1 Comment # 3

The reworked supplementary figures present the data more clearly. However, it is simply stated that there was no diurnal effect without any statistical analysis provided to qualify this. In Fig.S2 Dopamine certainly looks like it fluctuates with CSF collection time… numbers will be required to convince the reader of the author's claims

Response

We agree with the reviewer that to our claim should be supported by statistical analysis. CSF collected in the same 6 hours intervals (0:01 – 06:00, 06:01 – 12:00, 12:01 – 18:00, 18:01 – 24:00) were grouped for this purpose. Kruskal-Wallis test indicated no difference in CSF catecholamine levels between these intervals, neither in hip fracture patients (all p-values>0.5, n= 115) nor in the medical delirium patients (all p-values> 0.5, n=26). We have included this information in the supplementary figure legends (S1 and S2) and revised the manuscript text:

This section of the manuscript now reads (page 7, Results subheading Catecholamine interrelations and relations to age, gender and diurnal rhythm):

Acute hip fracture surgery and diagnostic LPs were performed at all hours but the CSF catecholamine levels did not differ in samples collected at different time intervals during the day (0:01 – 06:00, 06:01 – 12:00, 12:01 – 18:00, 18:01 – 24:00, Supplementary Fig.S1 and S2).

The revised Supplementary Figure legends now reads:

Supplementary Fig.S1: CSF catecholamine levels and time of day (time) of hip fracture surgery (time of lumbar puncture). Analyses of CSF samples collected within the same 6 hour intervals (0:01 – 06:00, 06:01 – 12:00, 12:01 – 18:00, 18:01 – 24:00) showed no significant differences in the CSF catecholamine levels (all p = 0.51, 0.72 and 0.81, Kruskal-Wallis test, n= 115).

Supplementary Fig S2: There were no diurnal fluctuations in the CSF catecholamine levels for the medical delirium patients. Analyses of CSF samples collected within the same 6 hour intervals (0:01 – 06:00, 06:01 – 12:00, 12:01 – 18:00, 18:01 – 24:00) showed no differences in the CSF catecholamine levels (all p = 0.78, 0.75 and 0.51, Kruskal-Wallis test, n= 26).

Reviewer 1 Comment #4

The revised section on CSF collection times, alongside the supplementary figures, is acceptable. However, 'daytime' is an unnecessarily vague term, please just state the time interval for the reader.

Response

We agree that we phrased this unnecessarily vague. Elective surgery is at our hospital performed between 0800 and 1700. We have examined the hospital records of all included patients and found only in one patient that surgery started outside this timeframe (at 21:00). This information is now included in the revised manuscript text (see below).

This section of the manuscript now read (page 5, Materials and Methods subheading CSF sampling and storage):

Elective surgeries were performed at daytime (0800-1700) and all the cognitively normal elderly patients except one (surgery started 2100) underwent surgery within this timeframe.

Reviewer 1 Comment #5

Gramma needs checking in many of the revised sections.

Reponse

We thank the reviewer for making us aware of this. Revisions are indicated by track changes in the enclosed manuscript file.

Reviewer 1 Comment #6

All other comments have been addressed. However, I am disappointed that the presence/absence of relevant dementia forms have not been confirmed, it would be surprising if this was not in the report/notes/data generated by the expert panel.

Response

We agree with the reviewer that information on dementia status is important in studies of delirium biomarkers. Since hip fracture is an acute event, objective cognitive testing prior to the fracture was not possible. Informant Questionnaire on Cognitive decline in the Elderly (IQCODE) is a recommended, validated and much used tool for assessing dementia status in such settings (Jackson et al., 2016), also in delirium studies of hip fracture patients (Juliebo et al., 2009; Witlox et al., 2011). The IQCODE does not give information on dementia forms.

In the OOT we followed the patients with cognitive tests (collected at home visits by research nurses) 12 months after surgery (MMSE, clock drawing test, 10-word list learning and recall from CERAD). Since these are objective cognitive tests, we believe use of these data could improve our precision of pre-fracture dementia status at baseline rather than relying on the IQCODE alone. All available information were therefore presented to two experts (including the IQCODE from baseline) who independently evaluated if the patients fulfilled the ICD10 criteria for dementia or not. We did not collect data on dementia forms, and this information is therefore missing from our dataset.

Details about the collection of cognitive data in OOT have been published earlier (Watne et al., 2014). If more detailed information regarding cognitive testing in the manuscript is desired, we would of course be happy to comply.

We have revised the manuscript to emphasize that information on dementia etiology is missing. This section of the manuscript now read (page 3-4, Methods, subheading Study participants):

During the hospital stay delirium was assessed daily and until the fifth post-operative day by the Confusion Assessment Method (CAM) (Inouye et al., 1990). Based on all available clinical information pre-fracture dementia status was determined by consensus in an expert panel. Dementia was diagnosed according to the International Classification of Diseases (ICD)-10 criteria (World Health Organization, 1993) without further information on dementia etiology. For further details see the previous publications (Wyller et al., 2012; Watne et al., 2014b).

Reviewer: 2
Comments to the Author

The study has been marginally improved, however concerns remain about the statistical rigor and study design, which were poorly rebutted by the authors.

Reviewer 2 Comment #1
In particular, the justification for not having power calculations as "the first study reporting CSF catecholamine levels in delirium" is unacceptable. Estimates can be calculated based on the changes herein provided. The exploratory nature of the study should not be presented as a valid justification for the "n" used here. Also, I don't think this is the first study ever measuring catecholamines in delirium (PMID: 32211614), thus data could likely be extrapolated and compared with other literature of CSF changes in delirium patients (not necessarily limited to catecholamines).

> Response:
> We agree with the reviewer that the report from Yasuda et al, examining serum noradrenaline in delirium, is relevant to discuss in our study. However, we disagree that we could use this report to calculate power. Firstly, at the time of publication of the report of Yasuda the catecholamine levels in the current study were already determined. Secondly, Yasuda and colleagues measured serum noradrenaline not CSF levels. CSF markers are considered to better reflect processes in the brain compartment than blood markers as substances may not cross the blood-brain-barrier (BBB). Correlations between CSF and serum levels might also be impacted by peripherally dilution, degradation and production (Blennow and Zetterberg, 2018). For noradrenaline correlations between plasma/serum have been reported but ranging from 0.3-0.8 (Borg *et al.*, 1982; Raskind *et al.*, 1984; Eldrup *et al.*, 1995) that add uncertainty to an extrapolation from the study of Yasuda and colleagues (n=81, of which 56 did not receive exogenous catecholamines). In conclusion it would not be straightforward to use the Yasuda study to estimate effect size in a CSF study even if this had been published prior to the study. Regarding power calculations please see the response to reviewer 1 comment # 2.
>
> We apologize that the study of Yasuda et al. not was included in the discussion of the current study results and are thankful to the reviewer for this reminder.
>
> The revised manuscript now reads (page 10-11, discussion):
>
> *CSF noradrenaline was higher in incident delirium patients without pre-fracture dementia but did not reach the significance level. This should be investigated in future studies as it suggest an increase in noradrenergic activity in the phase before delirium is clinically evident that support dexmedetomidine as a delirium preventive agent (Su et al., 2016). The medical delirium patients were all admitted to the hospital with ongoing delirium and CSF changes in the phase prior to clinically evident delirium could not be explored in these patients. However a recent study found serum noradrenaline levels associated with medical ICU-acquired delirium (Yasuda et al., 2020) suggesting that noradrenaline in delirium may be systemically elevated.*

Reviewer 2 Comment #2
The next major limitation is the cross-sectional approach. Not having baseline measurement, especially for catecholamines that are extremely sensitive to multiple pre-existing/pre-operative

stressors, cannot be justified by invoking ethical principles. Several studies are able to obtain lumbar CSF as part of research studies with necessary consent forms, IRB approval, and safety measures to these subjects. It sure is a lot of work, but is doable and done my several groups worldwide. Thus, I consider this is a major flaw in the study design that precludes in my opinion from a full evaluation of these results.

Response

We agree with the reviewer that baseline measurements of CSF catecholamines would have been very valuable in order to understand their potential role in delirium pathophysiology. Of studies exploring delirium pathophysiology, we know of only one study that have managed to collect repeated CSF samples (Hirsch *et al.*, 2016). In this study, Hirsch et al. collected repeated samples of CSF from 10 patients undergoing elective knee surgery. CSF was collected up to 24 hours after surgery through an indwelling spinal catheter inserted at the onset of spinal anesthesia. As expected with elective surgery, delirium incidence was low in this study, and only one patient developed postoperative delirium. Hip fracture patients in which delirium is a much more common complication (Bruce *et al.*, 2007), are unfortunately not well suited for such a study. Since a hip fracture is an acute event, in order to have a baseline sample, CSF would have to be collected from a cohort of patients before they broke their hip. This cohort would then had to be followed for years afterwards until a sufficient large proportion had experienced a hip fracture. Such a study could give fantastic information on delirium pathophysiology, but to our knowledge it has to date never been performed.

References:

Blennow, K., Zetterberg, H., 2018. The Past and the Future of Alzheimer's Disease Fluid Biomarkers. J. Alzheimers Dis. 62(3), 1125-1140.

Borg, J., Warter, J.M., Schlienger, J.L., Imler, M., Marescaux, C., Mack, G., 1982. Neurotransmitter modifications in human cerebrospinal fluid and serum during hepatic encephalopathy. J. Neurol. Sci. 57(2-3), 343-356.

Bruce, A.J., Ritchie, C.W., Blizard, R., Lai, R., Raven, P., 2007. The incidence of delirium associated with orthopedic surgery: a meta-analytic review. Int. Psychogeriatr. 19(2), 197-214.

Eldrup, E., Mogensen, P., Jacobsen, J., Pakkenberg, H., Christensen, N.J., 1995. CSF and plasma concentrations of free norepinephrine, dopamine, 3,4-dihydroxyphenylacetic acid (DOPAC), 3,4-dihydroxyphenylalanine (DOPA), and epinephrine in Parkinson's disease. Acta Neurol. Scand. 92(2), 116-121.

Faul, F., Erdfelder, E., Lang, A.-G., Buchner, A., 2007. G*Power 3: A flexible statistical power analysis program for the social, behavioral, and biomedical sciences. Behav. Res. Methods 39(2), 175-191.

Hirsch, J., Vacas, S., Terrando, N., Yuan, M., Sands, L.P., Kramer, J., Bozic, K., Maze, M.M., Leung, J.M., 2016. Perioperative cerebrospinal fluid and plasma inflammatory markers after orthopedic surgery. J. Neuroinflammation 13(1), 211.

Hoenig, J.M., Heisey, D.M., 2001. The Abuse of Power: The Pervasive Fallacy of Power Calculations for Data Analysis. The American Statistician 55(1), 19-24.

Jackson, T.A., MacLullich, A.M., Gladman, J.R., Lord, J.M., Sheehan, B., 2016. Diagnostic test accuracy of informant-based tools to diagnose dementia in older hospital patients with delirium: a prospective cohort study. Age Ageing 45(4), 505-511.

Juliebo, V., Bjoro, K., Krogseth, M., Skovlund, E., Ranhoff, A.H., Wyller, T.B., 2009. Risk factors for preoperative and postoperative delirium in elderly patients with hip fracture. J. Am. Geriatr. Soc. 57(8), 1354-1361.

Lydersen, S., 2015. Statistical review: frequently given comments. Ann. Rheum. Dis. 74(2), 323-325.

Nygaard, S., Christensen, A.H., Rolid, K., Nytrøen, K., Gullestad, L., Fiane, A., Thaulow, E., Døhlen, G., Godang, K., Saul, J.P., Wyller, V.B.B., 2019. Autonomic cardiovascular control changes in recent heart transplant recipients lead to physiological limitations in response to orthostatic challenge and isometric exercise. Eur. J. Appl. Physiol. 119(10), 2225-2236.

Pytte, M., Kramer-Johansen, J., Eilevstjønn, J., Eriksen, M., Strømme, T.A., Godang, K., Wik, L., Steen, P.A., Sunde, K., 2006. Haemodynamic effects of adrenaline (epinephrine) depend on chest compression quality during cardiopulmonary resuscitation in pigs. Resuscitation 71(3), 369-378.

Raskind, M.A., Peskind, E.R., Halter, J.B., Jimerson, D.C., 1984. Norepinephrine and MHPG levels in CSF and plasma in Alzheimer's disease. Arch. Gen. Psychiatry 41(4), 343-346.

Undrum Bergland, O., Larstorp, A.C.K., Lund Søraas, C., Høieggen, A., Rostrup, M., Norheim Kjaer, V., Godang, K., Sevre, K., Fadl Elmula, F.E.M., 2021. Changes in sympathetic nervous system activity after renal denervation: results from the randomised Oslo RDN study. Blood Press., 1-11.

Watne, L.O., Torbergsen, A.C., Conroy, S., Engedal, K., Frihagen, F., Hjorthaug, G.A., Juliebo, V., Raeder, J., Saltvedt, I., Skovlund, E., Wyller, T.B., 2014. The effect of a pre- and postoperative orthogeriatric service on cognitive function in patients with hip fracture: randomized controlled trial (Oslo Orthogeriatric Trial). BMC Med. 12, 63.

Witlox, J., Kalisvaart, K.J., de Jonghe, J.F., Verwey, N.A., van Stijn, M.F., Houdijk, A.P., Traast, H.S., MacLullich, A.M., van Gool, W.A., Eikelenboom, P., 2011. Cerebrospinal fluid beta-amyloid and tau are not associated with risk of delirium: a prospective cohort study in older adults with hip fracture. J. Am. Geriatr. Soc. 59(7), 1260-1267.

Yasuda, Y., Nishikimi, M., Nishida, K., Takahashi, K., Numaguchi, A., Higashi, M., Matsui, S., Matsuda, N., 2020. Relationship Between Serum Norepinephrine Levels at ICU Admission and the Risk of ICU-Acquired Delirium: Secondary Analysis of the Melatonin Evaluation of Lowered Inflammation of ICU Trial. Crit Care Explor 2(2), e0082.

Zhang, Y., Hedo, R., Rivera, A., Rull, R., Richardson, S., Tu, X.M., 2019. Post hoc power analysis: is it an informative and meaningful analysis? General Psychiatry 32(4), e100069.
