# Supplementary material for: Cerebrospinal fluid catecholamines in delirium and dementia
Source: Brain Commun. 2021 May 29;3(3):fcab121. doi: 10.1093/braincomms/fcab121 (PMC8374970; doi:10.1093/braincomms/fcab121)
Supplement: fcab121_Supplementary_Data [file Revision 2 – Decision Letter.pdf › Revision 2 – Decision Letter.pdf]

Revision 2 – Decision Letter

28-Apr-2021

Dear Dr. Henjum,

It is a pleasure to accept your manuscript entitled "Cerebrospinal fluid catecholamines in delirium and dementia" for publication in Brain Communications. You will see one of the reviewers has flagged a couple of grammatical errors which I hope you will correct at the first look stage.

The next step is for your paper to go through “First Look”. We will contact you again shortly if any final files or formatting changes are required for publication or if we have any queries.

If no changes are needed, your submission will be transferred to our Production Department and you will hear from the Production Editor in due course.

Video abstracts - Brain Communications encourages authors to provide a video abstract to accompany their article. These are hosted on the Brain/Brain Communications YouTube channel. If you are interested in producing a 5-min video abstract to promote your article, then please contact the Editorial Office (brcoms.editorialoffice@oup.com) for further details and guidelines.

Thank you very much for your contribution. On behalf of the Editors of Brain Communications, we look forward to your future contributions to the Journal.

Yours sincerely,

Professor Tara Spires-Jones
Founding Editor, Brain Communications

Reviewer(s)' Comments to Author:

Reviewer: 1

Comments to the Author
Changes to the methods section leave me no better able to judge the quality of the measurements made. For clarification, I am not in doubt that the kit and method used were appropriate. Measurement precision, as demonstrated by coefficient of variance, was decent. There is no information demonstrating the accuracy of measurements actually made in this study. This does not fundamentally undermine the study, I simply feel data quality should be well demonstrated and not assumed.

The authors have made no attempt to conduct a post-hoc power analysis to enable the reader to gauge the relevance of their data. They flag the issue in discussion of the study’s limitations in a manner that is, for better or worse, commonly accepted.

The authors support their claim that diurnal variation in surgery and sample collection time did not influence their data with a Kruskal-Wallace test, which seems appropriate. Honestly though, if the 13:00-14:00 spike in Fig.s2 Dopamine is not significant, I don’t know what would be.

Sample collection times are now presented in acceptable manner.

Grammar still needs checking e.g.

“All CSF samples available from this trial was included in the current study.”

“longitudinal CSF delirium studies is challenging to perform”
